# A Large-Aperture Variable Iris Directly Driven by a V-Shaped Mode-Coupled Ultrasonic Motor: Design, Optimization, and Experimental Validation

**DOI:** 10.3390/mi17091089

**Published:** 2026-09-16

**Authors:** Benchao Lv, Jun Zhao, Hongsheng Sun, Xucong Bao, Tong Zhao, Xiaolin Wang, Zhiyuan Yao, Xinjian Li, Xiaoniu Li

**Affiliations:** 1 National Key Laboratory of Infrared Detection Technologies, Shanghai 200083, China; 2 College of Aerospace Engineering, Nanjing University of Aeronautics and Astronautics, Nanjing 210016, China; 3 Kunming Physics Research Institute, Kunming 650223, China; 4 College of Automation, Nanjing University of Aeronautics and Astronautics, Nanjing 211106, China; 5 College of Marine Engineering and Electrical Engineering, Jiangsu Maritime Institute, Nanjing 211170, China

**Keywords:** mode-coupled ultrasonic motor, variable iris, direct drive, flexible clamping, compliant preload, aperture regulation

## Abstract

Large-aperture variable irises must provide continuous aperture regulation within a restricted axial space. This study presents a laboratory prototype of a 20-blade iris directly driven by a V-shaped mode-coupled ultrasonic motor. The iris was geometrically designed for a 5.5 mm–98 mm clear-aperture range. A wave-spring–bearing compliant preload mechanism was designed to maintain blade compression while permitting low-resistance actuating-ring rotation. Under a 3 N axial load, finite-element analysis showed that a representative three-blade overlap unit was approximately 7.6 times stiffer axially than the preload mechanism, so deformation occurred mainly in the latter. Taguchi design and multi-objective optimization produced a flexibly clamped stator with simulated working modes at 121.93 kHz and 122.10 kHz, separated by 170 Hz; the measured frequencies were 120.7 kHz and 121.5 kHz. At 121.8 kHz and 300 $V_{\mathrm{pp}}$, the 5.74 g stator generated bidirectional maximum thrusts of 3.91 N and 4.11 N, corresponding to a maximum thrust-to-mass ratio of 716 N/kg. A separate short-cycle test yielded a minimum observed stable tangential displacement of 3.23 μm. Quantitative opening and closing tests over a local 10 mm–20 mm interval yielded mean absolute actuating-ring angle errors of 0.087° and 0.13°, respectively. Model-extrapolation tests from a 20 mm reference yielded an aperture MAE of 0.183 mm over 10 mm–80 mm, while errors of 1.7 mm and −1.2 mm were measured at the 5.5 mm and 98 mm targets. These results support the functional feasibility of the proposed direct-drive architecture and identify an increased prediction error near the travel limits.

## 1. Introduction

Infrared detectors commonly employ a cold shield to suppress out-of-field stray radiation [1,2]. When the distance from the cold-shield opening to the focal plane is fixed, its effective aperture determines the detector F-number. During field-of-view or focal-length switching, unsynchronized F-number adjustment can cause throughput loss, vignetting, or additional stray radiation [1,2]. A variable cold iris must therefore regulate its clear aperture within limited axial space. Previous studies have shown that ultrasonic motors can operate in high and ultrahigh vacuums and can provide precision positioning [3,4]. However, vacuum alters frictional contact, wear, and heat dissipation, while temperature changes can affect output torque, speed, and power [5]. Ultrasonic motors therefore have potential as actuators for variable cold irises, provided that friction materials, outgassing, and temperature-induced drift are addressed in the design.

Existing aperture technologies include liquid irises or liquid lenses [6,7], multiblade mechanical irises [8], and piezoelectrically driven mechanisms [9]. Multiblade irises can form large, nearly circular apertures, but increasing their diameter enlarges the blade stack and friction load. Conventional geared transmission also adds axial components and assembly interfaces. Ultrasonic motors (USMs) offer compact construction, direct-drive capability, self-holding behavior, and immunity to electromagnetic interference [10,11,12,13].

To meet the requirements of compactness and direct drive, a variety of ultrasonic-motor configurations have been developed. Compact linear USMs based on flexural, symmetric, I-shaped, or submillimeter vibrators can drive local sliders and small loads [14,15,16,17]. Rotary, multi-vibrator, stacked, and multi-degree-of-freedom configurations extend the available output motions [18,19,20,21,22,23,24]. However, these studies do not directly address peripheral actuation of a large annular member within a thin optical envelope. For this application, the motor–load interface and axial transmission path are as important as the motor dimensions.

Reversible single-tip friction drive requires two near-frequency working modes to provide tangential and normal vibration components that combine into a stable elliptical trajectory at the driving tip. Consequently, working-mode frequency matching and the preservation of the driving-tip amplitudes under the actual clamping boundary are central to stator design. Longitudinal–bending coupled transducers and linear motors have been analyzed using transfer matrices, finite-element analysis (FEA), and experiments [25,26,27]. Taguchi design can reduce the initial geometry space for matching two working modes [28]. Power-flow and driving-tip optimization studies further show that energy-transfer paths and local geometry affect output performance [29,30]. The actual clamping boundary also shifts the working modes and driving-tip amplitudes. Stator design must therefore consider frequency matching, bidirectional tip motion, adjacent-mode isolation, stiffness, and manufacturability together.

Our group previously used an annular traveling-wave rotary USM to drive an iris with a 4 mm–30 mm aperture range [31]. Motion was transmitted to the iris actuating ring through a separate motor rotor and connecting ring, which stacked the motor and iris components along the optical axis and increased the axial dimension and number of assembly interfaces. As the aperture increases, the rotor and connecting ring must also increase in size, which is unfavorable for thin, integrated large-aperture irises. To shorten the transmission chain, the present design places a localized V-shaped stator outside the clear aperture and applies the driving tip directly to the outer edge of the actuating ring.

To address these limitations, this study develops a 20-blade large-aperture variable iris peripherally driven by a V-shaped mode-coupled USM. The main work comprises: (1) an outer-edge direct-drive architecture and a wave-spring–bearing compliant preload mechanism that decouples axial blade compression from circumferential actuating-ring rotation; (2) a two-stage design method combining Taguchi screening and clamped-boundary multi-objective optimization for working-mode matching, amplitude optimization, and adjacent-mode isolation; and (3) electrical, vibration, load, microstepping, and temperature characterization of the stator and motor, followed by integration, local aperture regulation, and full-range model-extrapolation tests of the 20-blade iris. Together, these tasks establish and experimentally validate a compact peripheral direct-drive architecture for large-aperture variable irises.

## 2. Design of the Large-Aperture Variable Iris and Compliant Preload Mechanism

### 2.1. Design Requirements and Overall Architecture

For a detector with a cold-shield-to-focal-plane distance, *f*, and effective cold-shield aperture, *D*, the *F*-number is

(1) $$F=\frac{f}{D}.$$

When *f* is fixed, the continuous regulation of *D* changes the detector *F*-number. The mechanism studied here was required to provide a large continuous aperture range while minimizing axial thickness. The optomechanical constraints specified a maximum aperture of 98 mm, a minimum aperture of 5.5 mm, 20 blades, and an overall envelope of 170 mm × 170 mm × 33.5 mm. Because the actuating ring has a large diameter and the available axial space is restricted, the driving tip contacts the outer edge of the ring directly, without a gear reducer.

**Figure 1:**
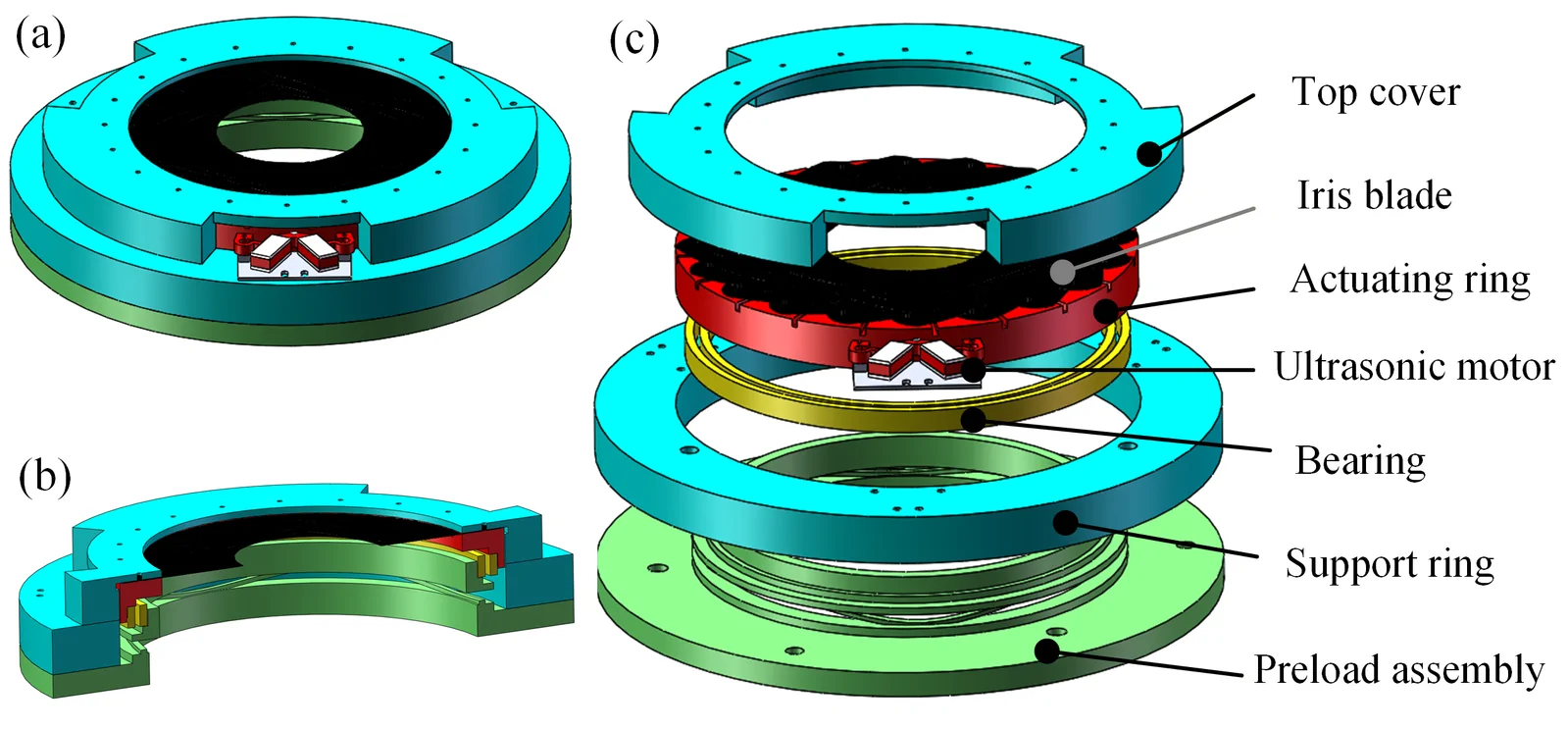
Large-aperture variable iris directly driven by the V-shaped mode-coupled USM: (**a**) overall view; (**b**) sectional view; and (**c**) exploded view.

The variable iris consists of a top cover, a fixed ring, 20 blades, an actuating ring, thin-section bearing, compliant preload mechanism, and V-shaped mode-coupled USM, as shown in Figure 1. A fixed pin at one end of each blade engages a cylindrical hole in the top cover, while a moving pin at the other end enters a guide slot in the actuating ring. The rotation of the ring moves the 20 pins synchronously along their slots, causing the blades to rotate about their fixed pins and form a continuously variable central aperture. The fixed ring supports the USM and constrains blade motion, while the top cover and fixed ring form a thin enclosed frame.

Two load paths are decoupled in the system. In the circumferential drive path, the piezoelectric ceramics convert high-frequency voltage into stator vibration. The two working modes produce an elliptical trajectory at the driving tip, whose frictional action rotates the actuating ring and drives the blades through the guide slots. In the axial preload path, a wave spring generates an axial force that is transmitted through the bearing to the actuating ring and blade stack. Relative motion between the bearing races allows circumferential rotation under axial load. The architecture therefore separates rotation transmission from normal-load application rather than simply adding a spring that would directly impede ring rotation. The principal design requirements and implemented prototype parameters are summarized in Table 1.

### 2.2. Blade Geometry and Conversion of Iris Requirements to Actuator Requirements

**Figure 2:**
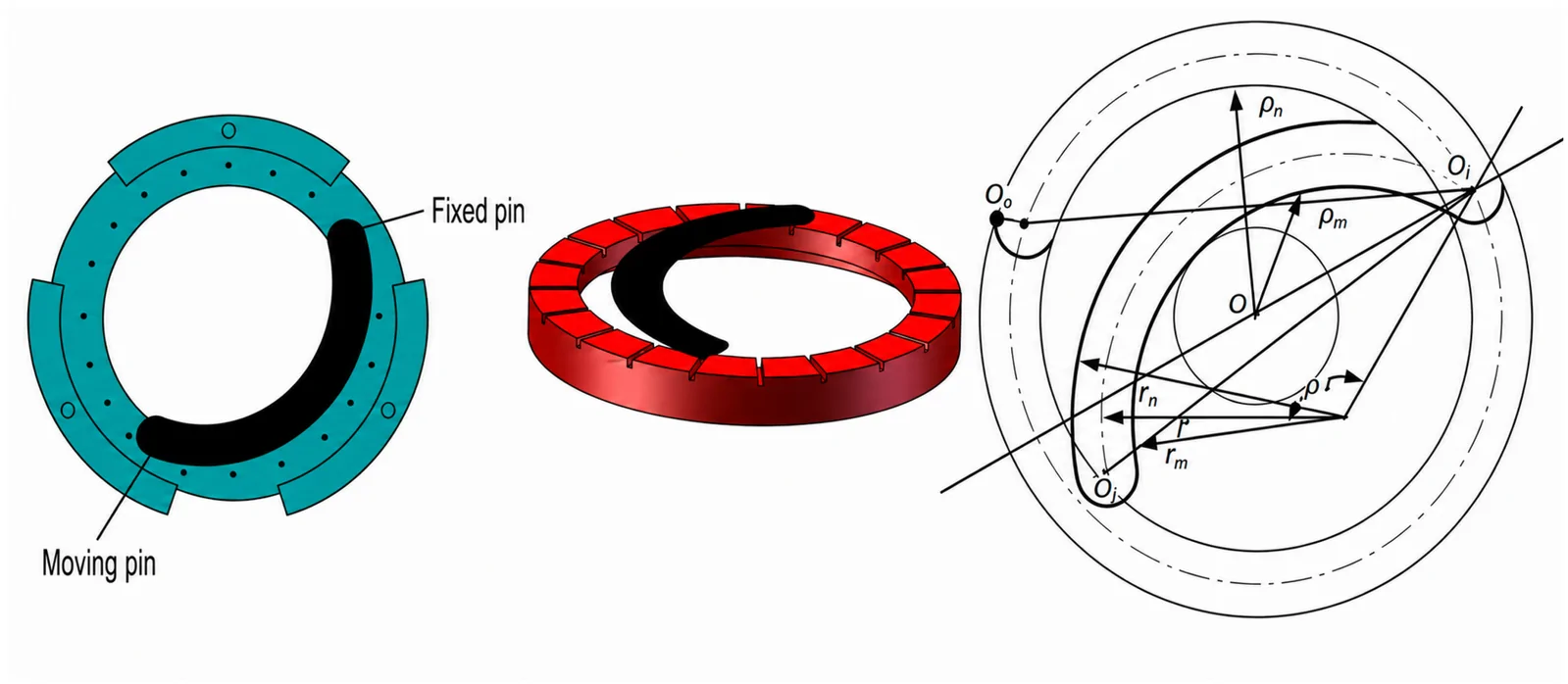
Blade–actuating-ring assembly and motion range of one blade.

Each 0.08 mm-thick stainless-steel blade has a double-arc profile, a fixed pin at one end, and a moving pin at the other. The double-arc geometry preserves overlap between adjacent blades at different openings and forms a nearly circular central aperture. Figure 2 shows the blade–ring assembly and the motion range of one blade. The left part illustrates the fixed pin, moving pin, and ring guide slot; the right part shows the geometric envelope from the maximum to the minimum opening.

Let the fixed pin of blade *i* lie on a circle of radius $r_{f}$ with initial polar angle $\varphi_{i}$. The moving pin lies near the circle constrained by the guide slot, and the actuating ring rotates through θ from its initial position. The fixed- and moving-pin positions can be expressed as

(2) $$P_{i}=r_{f}{\left[\cos\varphi_{i},\;\sin\varphi_{i}\right]}^{T},\;Q_{i}\left(\theta \right)=r_{m}{\left[\cos\left(\varphi_{i}+\theta \right),\;\sin\left(\varphi_{i}+\theta \right)\right]}^{T}.$$

The geometric constraint between the two pins determines the blade orientation $\alpha_{i}\left(\theta \right)$, and the minimum envelope of all inner blade arcs defines the effective aperture. The aperture is therefore a single-valued function of the actuating-ring angle,

(3) $$D=G(\theta ;r_{f},r_{m},r_{n},r_{m}^{\prime },l_{p},N_{b}),$$

where $r_{n}$ and $r_{m}^{\prime }$ are geometric parameters of the outer and inner blade arcs, $l_{p}$ is the pin-to-pin distance, and $N_{b}=20$ is the number of blades. Analytical geometry gave a 141.21° included angle between the two pins about the blade center and a minimum required overlap count of 19.25; rounding upward gave 20 blades. This mapping was used both to verify the attainable 5.5 mm–98 mm aperture range and to convert tangential driving-tip displacement into an aperture increment.

At an effective ring contact radius, $R_{d}$, the tangential driving-tip displacement and ring angle approximately satisfy $s_{t}=R_{d}\theta$. A smaller motor step permits a smaller angular increment, whereas motor thrust must overcome bearing resistance, tip-contact losses, and interblade friction. Direct-drive feasibility was evaluated through bidirectional maximum-thrust measurements and integrated functional tests.

The blade-pin circle had a radius of 55.3 mm. Together with the 141.21° included angle and the double-arc geometry, this pin arrangement maintained overlap among all 20 blades over the full designed travel.

### 2.3. Compliant Preload Mechanism and Stiffness Analysis

**Figure 3:**
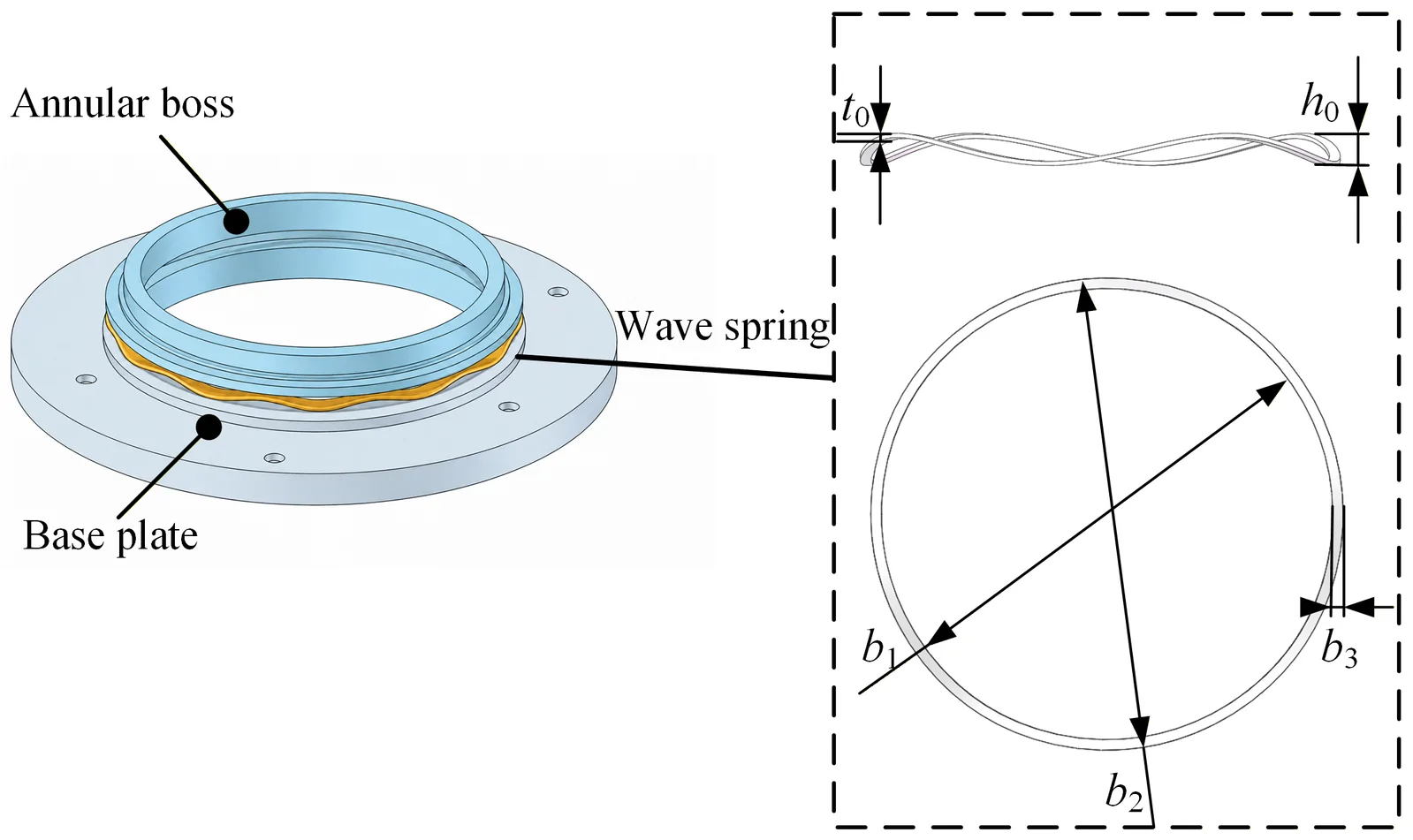
Wave-spring preload assembly and its principal geometric parameters.

The number of overlapping blade layers changes with aperture and produces a small axial displacement of the actuating ring relative to the fixed ring. If a rigid pressure plate applies the load, this displacement can cause a large change in normal force and consequently amplify blade friction. A thin-section bearing was therefore placed between the actuating ring and the preload mechanism. The outer bearing race engages the annular boss on the actuating ring, while the inner race contacts the boss on the preload mechanism. The axial force generated by the wave spring is transmitted to the ring through the bearing, and rolling motion releases the ring rotation, as shown in Figure 3.

Following the load–displacement model developed by Dragoni for annular wave springs [32], the axial reaction of the single-layer spring was written under a small-deformation approximation as

(4) $$F_{b}=\frac{16K_{1}N_{w}^{4}b_{3}t_{0}^{3}E_{1}}{\pi^{3}b_{4}^{3}}s,$$

where

(5) $$b_{4}=\frac{b_{1}+b_{2}}{2}.$$

Here, $F_{b}$ is the axial reaction force; $K_{1}$ is a geometric correction factor; $N_{w}$ is the number of waves; $b_{1}$, $b_{2}$, and $b_{4}$ are the inner, outer, and mean diameters; $b_{3}$ is the annular-band width; $t_{0}$ is the thickness; $E_{1}$ is Young’s modulus; and *s* is the axial compression. Table 2 lists the parameters selected from the approximately 3 N target preload and available radial space. The preload body was modeled as polytetrafluoroethylene with a Poisson’s ratio of 0.4 and a Young’s modulus of $2.8\times 10^{-8}$ Pa.

The stiffness comparison used direct axial loading and omitted sliding contact. Interblade friction and bearing resistance were represented by equivalent parameters in the integrated system model described in Section 5.

**Figure 4:**
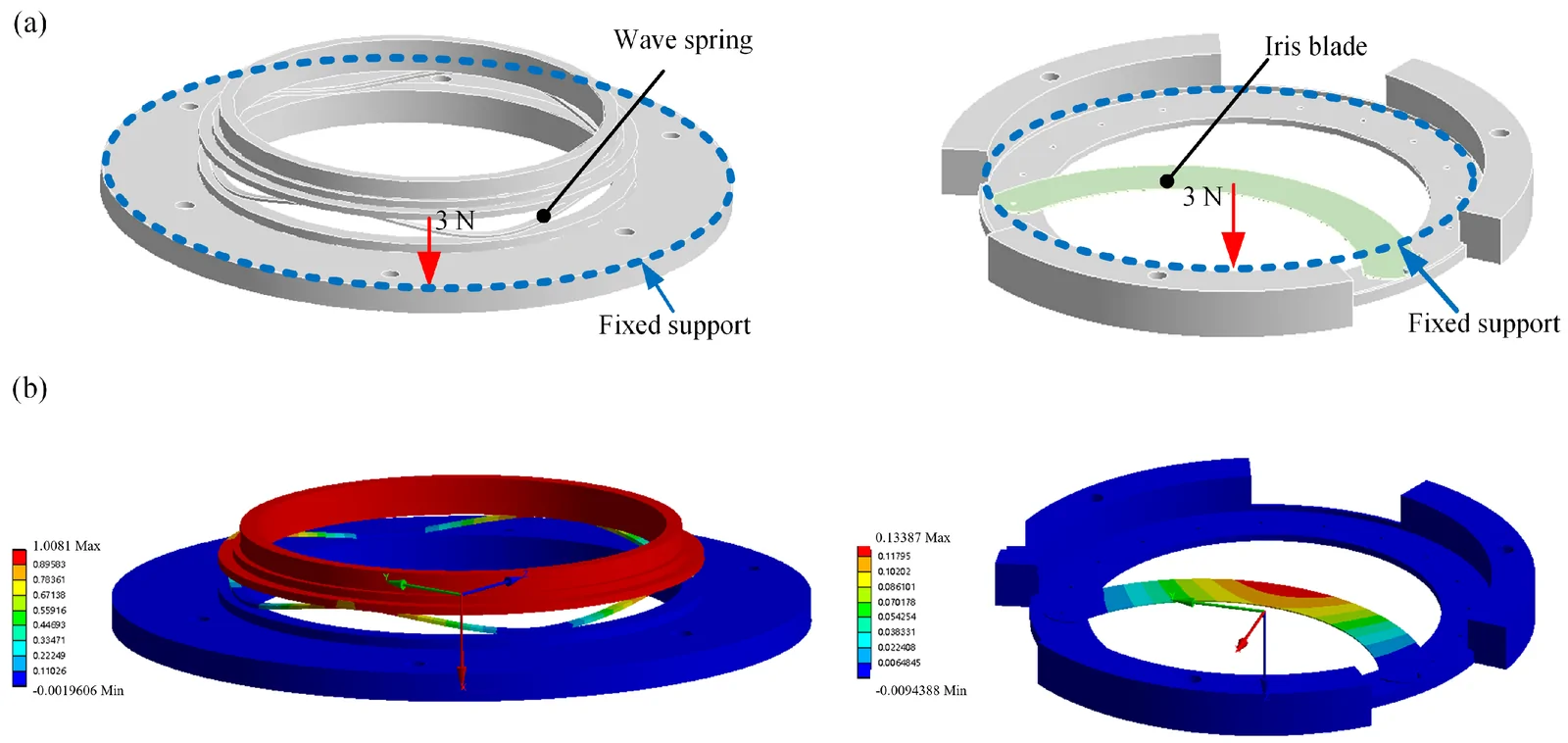
Finite-element stiffness comparison under identical 3 N axial loads: (**a**) boundary conditions for the compliant preload mechanism (**left**) and the representative three-blade overlap unit (**right**), where blue dashed boundaries denote fixed supports and red arrows denote the applied loads; (**b**) axial directional-deformation contours of the preload mechanism in the *x*-direction (**left**) and the blade unit in the *z*-direction (**right**). The color legends range from minimum deformation (blue) to maximum deformation (red); the maximum values are 1.0081 mm and 0.13387 mm, respectively.

The design criterion was that the equivalent stiffness of the preload mechanism be lower than that of the blade stack. Let $k_{p}$ and $k_{b}$ denote the corresponding stiffnesses. When $k_{p}\ll k_{b}$, axial motion induced by changes in blade overlap is accommodated mainly by the preload mechanism, reducing variations in blade normal force and friction. Separate finite-element models were established for the preload mechanism and a representative unit of three fully overlapping blades. The three-blade unit represents the local fully overlapped region used for controlled stiffness comparison, whereas the stiffness of the complete stack varies with aperture. The mounting surface of the preload base and the blade support locations were fixed, and a 3 N axial load was applied to each load-bearing surface. Figure 4 shows the boundary conditions and deformation contours, and Table 3 compares the calculated deformation and equivalent stiffness.

The FEA-predicted stiffness ratio $k_{b}/k_{p}$ was approximately 7.6, indicating that the compliant preload mechanism accommodates most of the blade-stack-induced axial displacement in this simplified model. Over the designed preload displacement range of 0 mm–0.85 mm, the calculated reaction remained approximately 3.0 N ± 0.1 N. These numerical results support the structural concept and parameter selection used in the prototype.

## 3. Design and Optimization of the V-Shaped Mode-Coupled Ultrasonic Motor

### 3.1. Stator Architecture and Operating Principle

**Figure 5:**
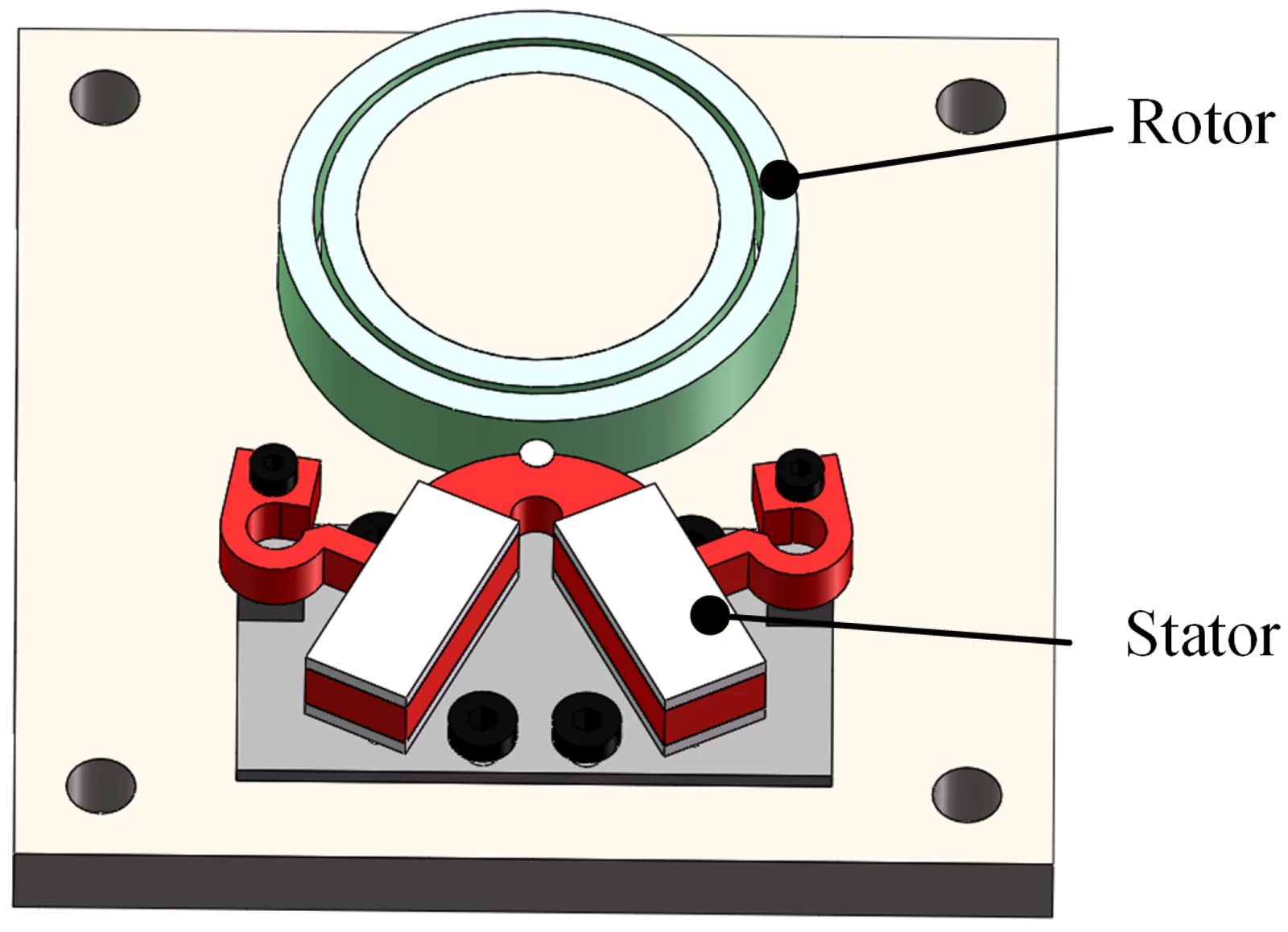
Contact architecture of the V-shaped stator and annular test rotor.

Figure 5 shows the contact arrangement between the V-shaped stator and the annular test rotor. Before integration with the iris, the annular rotor was used as a test load to reproduce frictional contact between the driving tip and the outer edge of the iris actuating ring. In the integrated system, the driving tip acts directly on the actual actuating ring. The stator assembly consists of a V-shaped metallic substrate, zirconia driving tip, and mounting base. The metallic substrate was machined from 7075 aluminum alloy. Two PZT-8 plates (DM-4D, Zhongshan DM Piezoelectric Ceramic Material Co., Ltd., Zhongshan, China) were bonded to each of its upper and lower surfaces; opposing plates on the upper and lower surfaces were polarized in opposite through-thickness directions. Each ceramic plate measured 14.5 mm × 6.5 mm × 1 mm. A cylindrical hole at the stator apex received the 2 mm-diameter zirconia driving tip. Manufacturing the tip separately from the aluminum substrate exploited the greater hardness and wear resistance of zirconia at the friction interface.

The stator uses one symmetric and one antisymmetric working mode. The symmetric mode is coupled from in-phase longitudinal vibration of the two V-shaped branches, whereas the antisymmetric mode is coupled from their out-of-phase longitudinal vibration. Because the branches are mirror-symmetric about the structural center plane, these coupled states provide different displacement components at the driving tip. Sinusoidal voltages of equal frequency and amplitude, with a 90° phase difference, are applied to phases A and B to superpose the two modes. If the tangential and normal tip displacements are approximated by

(6) $$u_{t}\left(t\right)=A_{t}\sin\left(\omega t\right),\;u_{n}\left(t\right)=A_{n}\cos\left(\omega t\right),$$

then the trajectory satisfies

(7) $$\frac{u_{t}^{2}}{A_{t}^{2}}+\frac{u_{n}^{2}}{A_{n}^{2}}=1.$$

**Figure 6:**
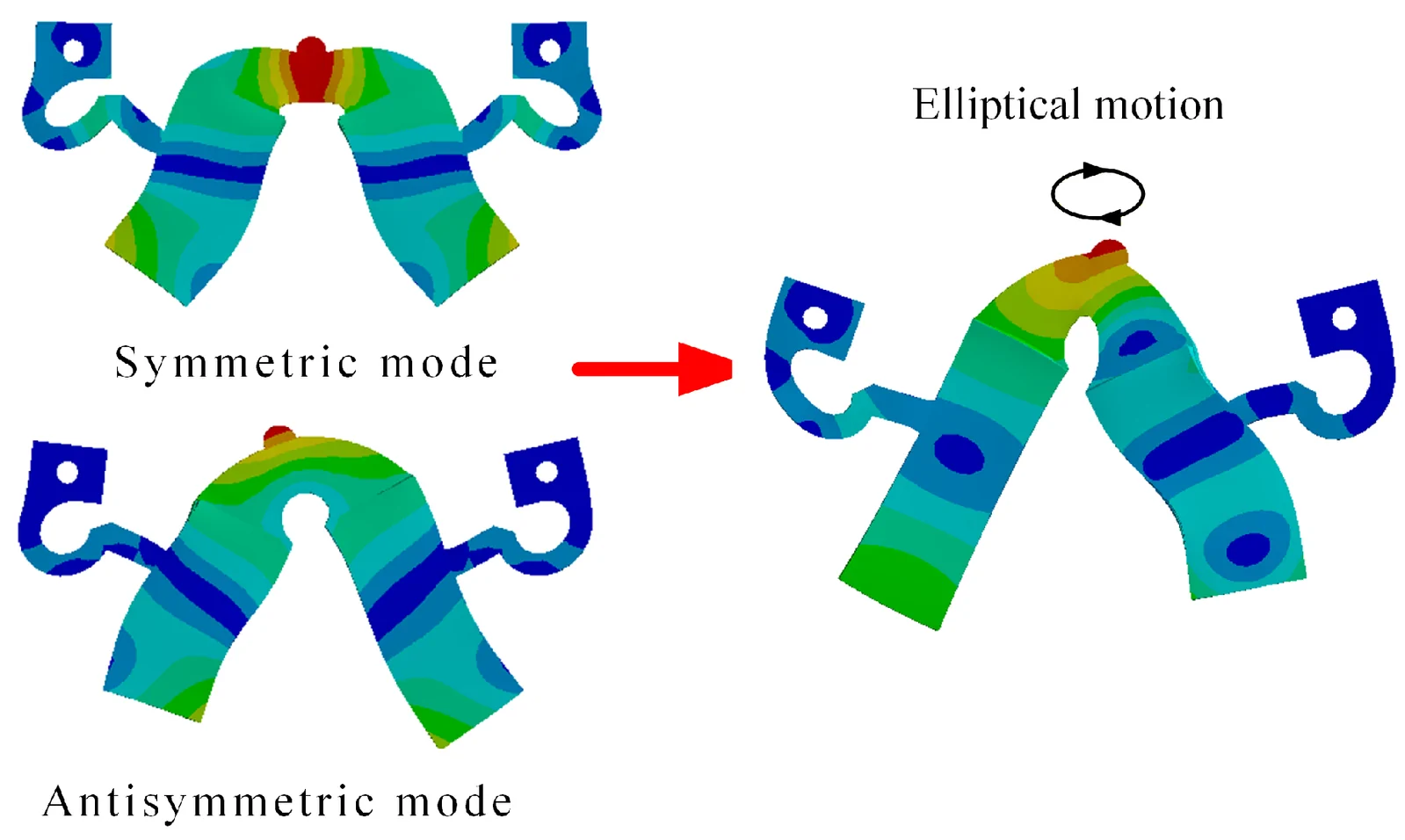
Coupling of the symmetric and antisymmetric modes to generate elliptical driving-tip motion. The color contours represent relative deformation magnitude.

The normal component periodically modulates contact, while the tangential component transfers friction force to the actuating ring during contact. Reversing which phase leads changes the direction of the elliptical trajectory and consequently switches between iris opening and closing. Figure 6 illustrates the two modes and their coupled motion at the driving tip.

### 3.2. Working-Mode Matching of the Unclamped Stator

The flexible clamps and metallic substrate were designed as a monolithic component; however, optimizing both simultaneously would obscure working-mode identification. A Taguchi orthogonal array was first used to screen the unclamped-stator design space [28]. The low-vibration region near the symmetric-mode node was then used to locate the clamps and provide identifiable initial modes for subsequent clamped-stator optimization. The unclamped-stator variables were $L_{1}$, $L_{2}$, $R_{1}$, $R_{2}$, and thickness *T*. Five levels were assigned to each variable, and 25 finite-element simulations were arranged using an $L_{25}\left(5^{5}\right)$ orthogonal array. The response was the absolute frequency difference between the symmetric and antisymmetric modes,

(8) $$\Delta f=\left|f_{s}-f_{a}\right|,$$

and the array was followed by an independent confirmation analysis of the retained parameter combination.

**Figure 7:**
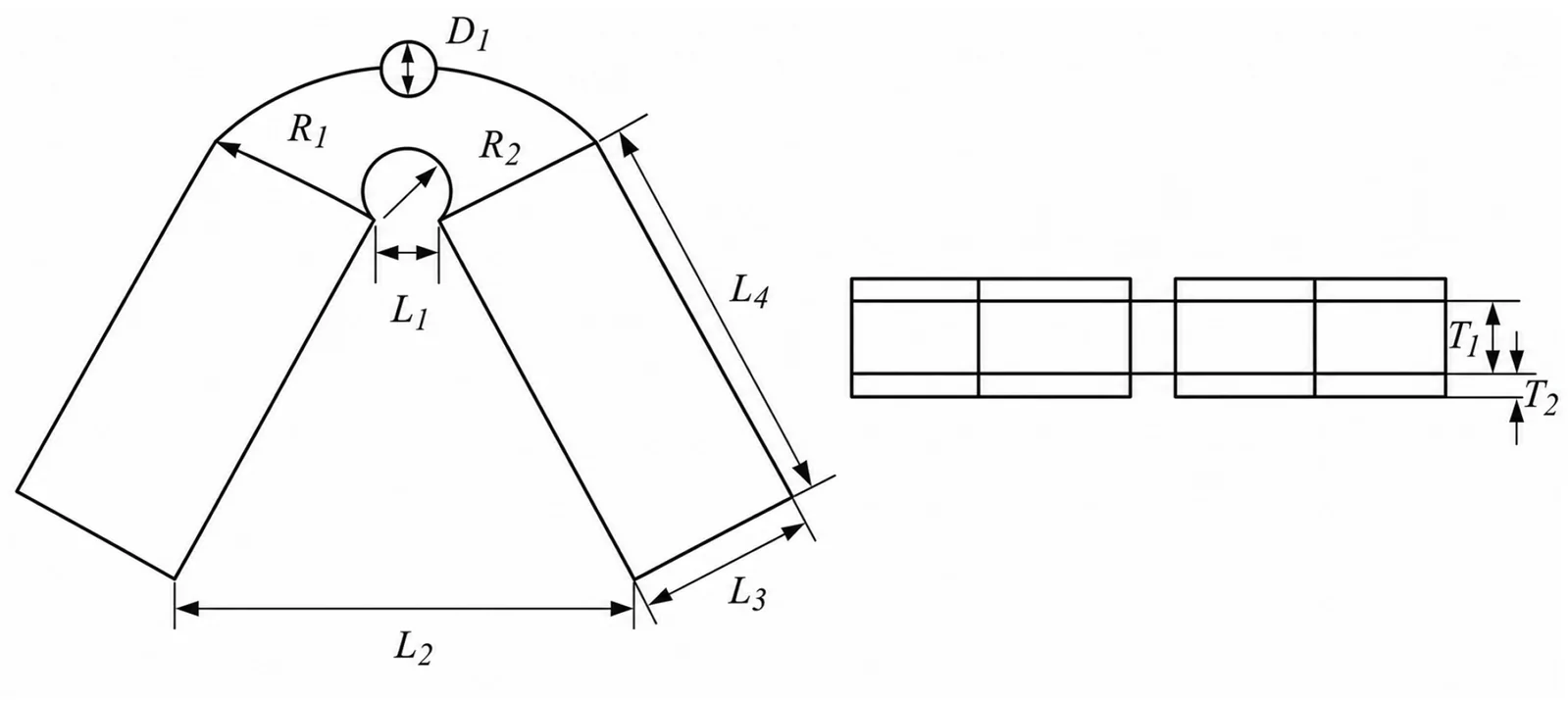
Geometry of the unclamped V-shaped stator and parameters used in the Taguchi design.

Figure 7 defines the geometric parameters, and Table 4 lists the factors and levels. All levels were separated by 0.1 mm; $L_{1}$, $L_{2}$, $R_{1}$, $R_{2}$, and *T* span manufacturable local lengths, overall lengths, radii, and thicknesses. The piezoelectric-plate dimensions and the 2 mm-diameter driving tip were fixed during this stage.

Table 5 lists the $L_{25}\left(5^{5}\right)$ orthogonal array and the 25 finite-element responses. The unclamped-stator model was rebuilt for every parameter set. Because the absolute working-mode frequency difference Δf was the sole response at this stage, $f_{s}$ and $f_{a}$ are not listed separately for each run.

Across the 25 design points, Δf ranged from 350 Hz to 2160 Hz. The retained combination was $L_{1}=2.1$ mm, $L_{2}=15.3$ mm, $R_{1}=9.5$ mm, $R_{2}=1.5$ mm, and T=3.3 mm. A separate confirmation model gave symmetric- and antisymmetric-mode frequencies of 121.77 kHz and 122.08 kHz, respectively, with Δf=310 Hz; the confirmation result is included as the final row of Table 5. The low-vibration region near the symmetric-mode node also provided the basis for locating the flexible clamps.

### 3.3. Flexible Clamping and Multi-Objective Amplitude Optimization

**Figure 8:**
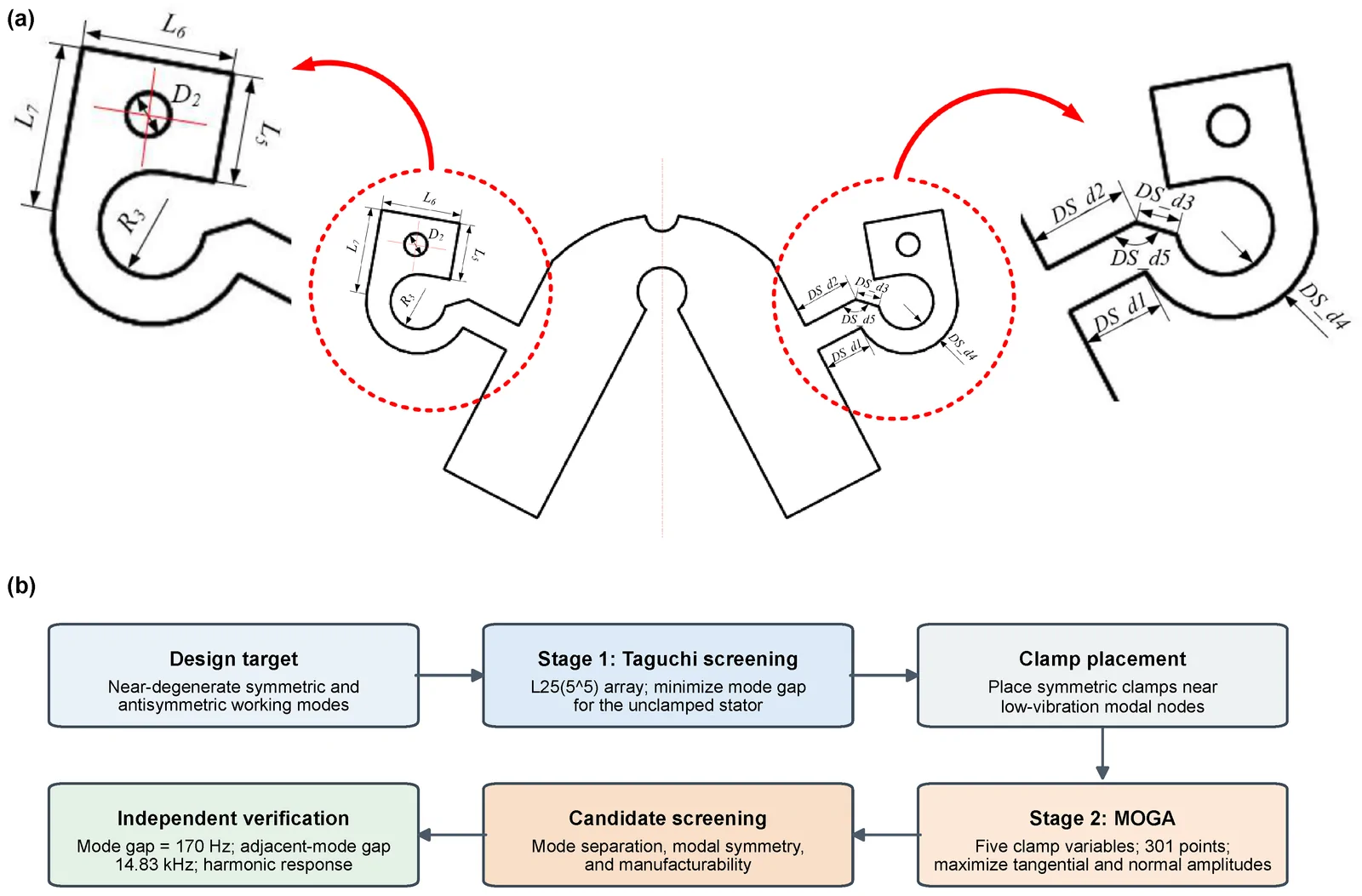
Flexible-clamp design: (**a**) geometry and design variables; (**b**) two-stage optimization workflow.

Symmetric curved flexible clamps were placed on both sides of the stator near the symmetric-mode nodes. The elastic deformation of the curved beams both fixes the stator and contributes to an adjustment of the driving-tip contact preload. Figure 8a defines the clamp region and local dimensions. Figure 8b summarizes the complete two-stage design procedure, from unclamped frequency matching to clamped-boundary optimization and independent verification.

Because the clamp parameters alter the modal frequencies, driving-tip amplitudes, and static stiffness simultaneously, the second-stage optimization did not minimize frequency difference alone. The maximum tangential and normal amplitudes of the driving tip were maximized, with lower-bound constraints of 100 μm for both responses. Five clamp parameters, $d_{1}$–$d_{5}$, were defined as design variables in ANSYS Workbench 2022 R1. Their ranges were 2.7 mm to 3.3 mm, 3.0 mm to 3.8 mm, 1.3 mm to 1.6 mm, 1.4 mm to 1.6 mm, and 135.5°–136.0°, respectively. The linear dimensions were discretized in 0.1 mm increments to reflect machining capability.

The direct-optimization module used a multi-objective genetic algorithm. Seven batches of 43 design points were evaluated, comprising one initial batch and six subsequent batches. The resulting 301 design points were screened, and three candidates were retained. Candidate 1 provided the highest predicted amplitude but only a 3.06 kHz minimum separation from an adjacent mode. Candidate 2 increased this separation to 11.69 kHz, but some local dimensions approached machining limits and the nominally symmetric mode was imperfectly symmetric. Candidate 3 gave a 170 Hz working-mode frequency difference and a 14.83 kHz minimum separation from adjacent parasitic modes while retaining better manufacturability. Candidate 3 was therefore selected instead of maximizing a single amplitude metric. The design variables and predicted amplitudes of the three candidates are listed in Table 6, and their modal verification results are summarized in Table 7.

The archived verification model of candidate 3 contained 26,859 nodes and 11,613 solid elements. Coupled-field elements represented the PZT domains, while structural solid elements represented the metallic and ceramic components. The harmonic response was calculated from 119 kHz to 122.55 kHz using 150 frequency substeps. The two phase electrodes were assigned 200 V harmonic-potential components in quadrature. The archived model omitted contact and explicit damping; the resulting resonance amplitudes were therefore used as relative metrics for candidate screening. All candidates were evaluated under the same numerical settings.

A separate harmonic-response verification of candidate 3 produced maximum tangential and normal responses of 680 μm and 557 μm, respectively. These values were close to the optimization outputs of 705.8 μm and 576.7 μm. Candidate 3 was subsequently evaluated through the thrust and motion experiments described in Section 4.

## 4. Motor Performance Characterization

### 4.1. Prototype Fabrication and Test Objects

**Figure 9:**
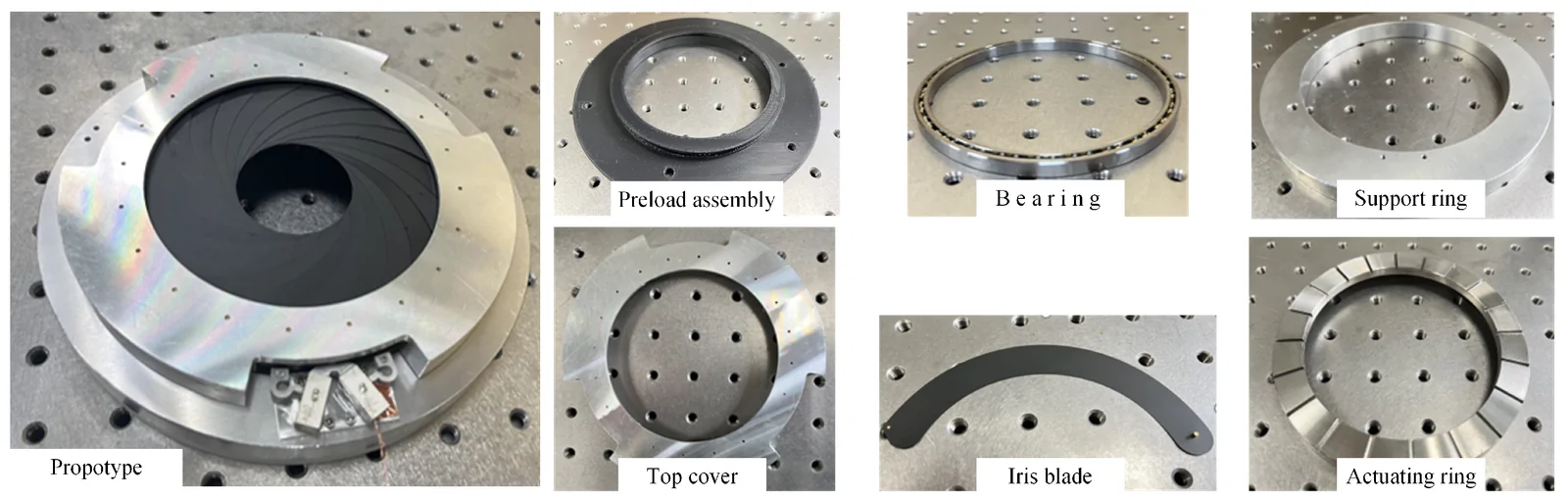
Prototype and principal components of the 20-blade variable iris directly driven by the V-shaped mode-coupled USM.

The metallic stator substrate was machined from 7075 aluminum alloy. The lead zirconate titanate (PZT-8) plates and zirconia driving tip were bonded to the substrate using 3M DP460 epoxy (3M Company, St. Paul, MN, USA). The assembled stator measured 36.6 mm × 19.8 mm × 5.3 mm, including the two 1 mm-thick PZT layers bonded to the 3.3 mm-thick metallic substrate, and had a mass of 5.74 g. The actuating ring was fabricated from 304 stainless steel, while the top cover and fixed ring were made of aluminum alloy. An HXHV KA042CP0 thin-section bearing (Wuxi HXH Bearing Co., Ltd., Wuxi, China), with an inner diameter of 107.95 mm, an outer diameter of 120.65 mm, and a thickness of 6.35 mm, was used. The assembled prototype occupied an envelope of 170 mm × 170 mm × 33.5 mm. Figure 9 shows the prototype and its principal components.

### 4.2. Electrical and Vibration Characteristics

#### 4.2.1. Electrical Characterization

**Figure 10:**
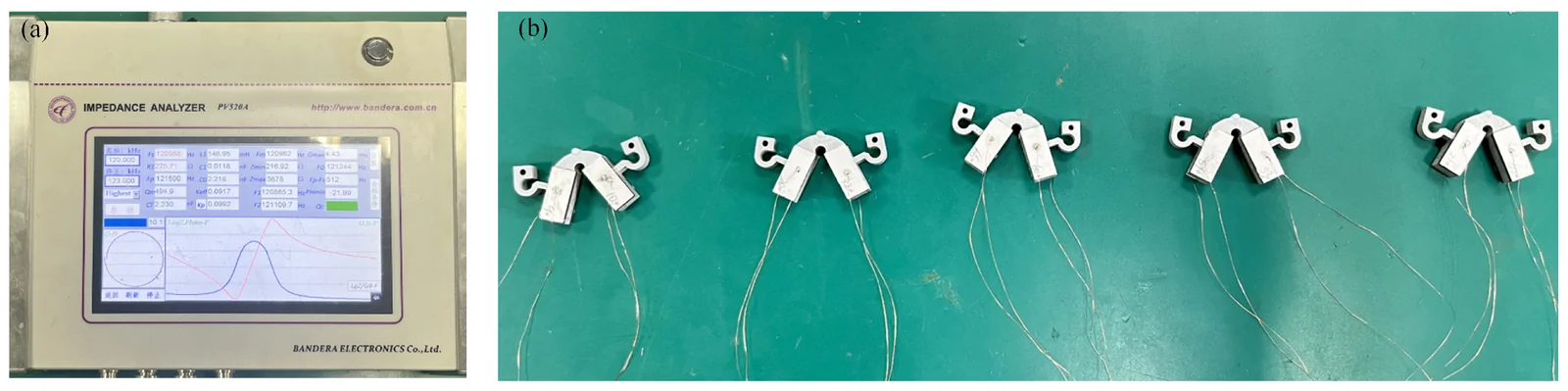
Electrical characterization of the stators: (**a**) PV520A impedance analyzer; and (**b**) representative tested stators. The Chinese labels visible on the analyzer screen are instrument-interface labels for the displayed electrical measurements; the quantities used in this study are defined in the text and reported in Table 8.

A PV520A impedance analyzer (Bandera Electronics Co., Ltd., Beijing, China) was used to measure the electrical characteristics of ten stators. Figure 10 shows the instrument and samples. The sweep range was 118 kHz–125 kHz. The analyzer ground was connected to the common stator electrode, while the measurement terminal was connected in turn to phases A and B. The static capacitance $C_{0}$, series-resonance frequency $F_{s}$, and parallel-resonance frequency $F_{p}$ were recorded.

The static capacitances of both phases were concentrated near 2.2 nF; the 20 measurements ranged from 2.113 nF to 2.286 nF. The two phases of each stator generally exhibited similar static capacitances and resonance frequencies. Stator 4 showed a comparatively large phase-to-phase capacitance difference, with 2.189 nF for phase A and 2.286 nF for phase B. This deviation may originate from machining error, adhesive-layer thickness, or solder-joint condition. The representative ranges are summarized in Table 8. Overall, the measurements indicate stable and consistent electrical characteristics among the fabricated stators.

#### 4.2.2. Vibration Characterization

**Figure 11:**
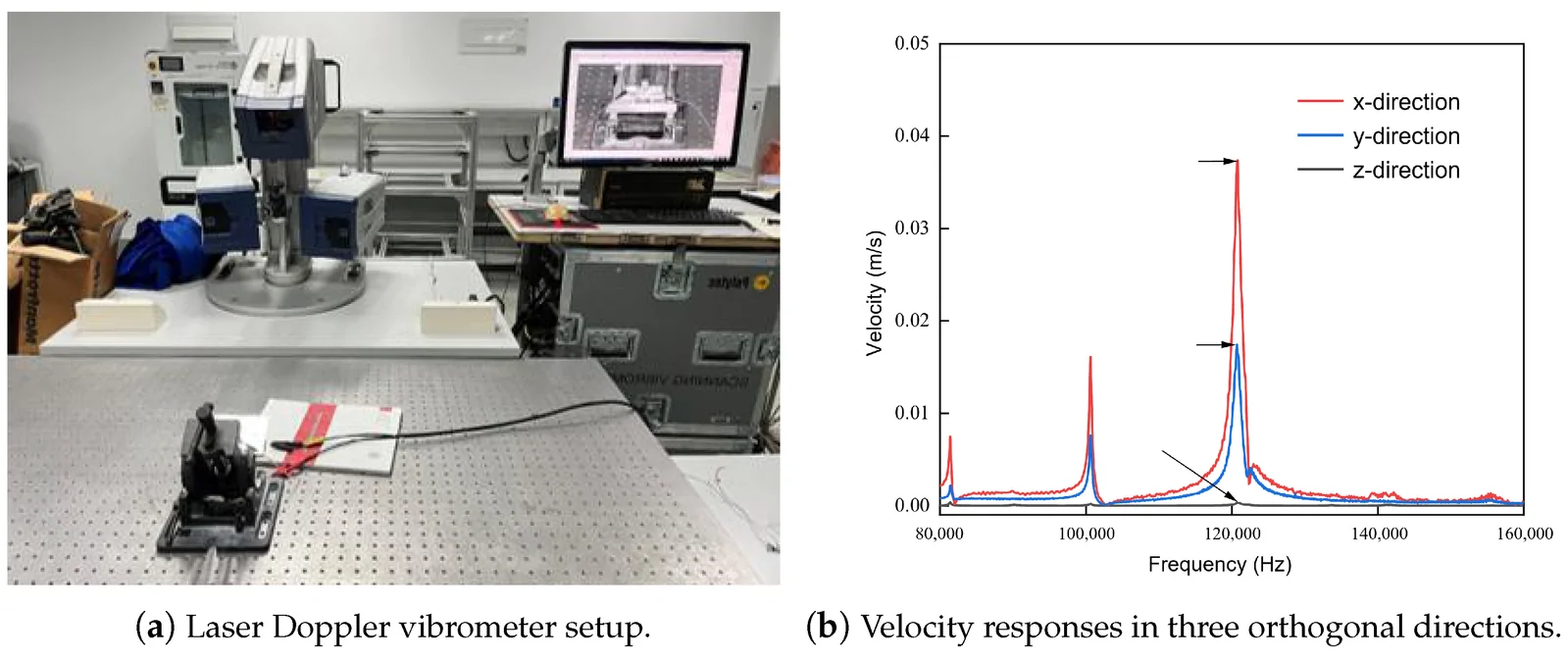
Stator vibration characterization: (**a**) experimental setup; and (**b**) measured frequency responses under 100 $V_{\mathrm{pp}}$ single-phase excitation.

A PSV-300F-B laser Doppler vibrometer (Polytec GmbH, Waldbronn, Germany) was used to measure stator vibration. A single phase was driven at 100 $V_{\mathrm{pp}}$; multiple measurement points were placed near the driving tip, and frequency sweeps spanning the working range provided velocity responses in three orthogonal directions. The test setup and measured responses are shown in Figure 11.

Table 9 compares the measured and simulated working-mode frequencies. The symmetric and antisymmetric modes were measured at 121.5 kHz and 120.7 kHz, corresponding to deviations of 0.5% and 1.0% from the finite-element values. The measured mode separation was 800 Hz, below the design allowance of 1 kHz but larger than the simulated 170 Hz. The model therefore predicted the working-frequency range, whereas the mode separation remained more sensitive to piezoceramic bonding, geometric tolerances, and the actual clamping boundary.

### 4.3. Load Characteristics

**Figure 12:**
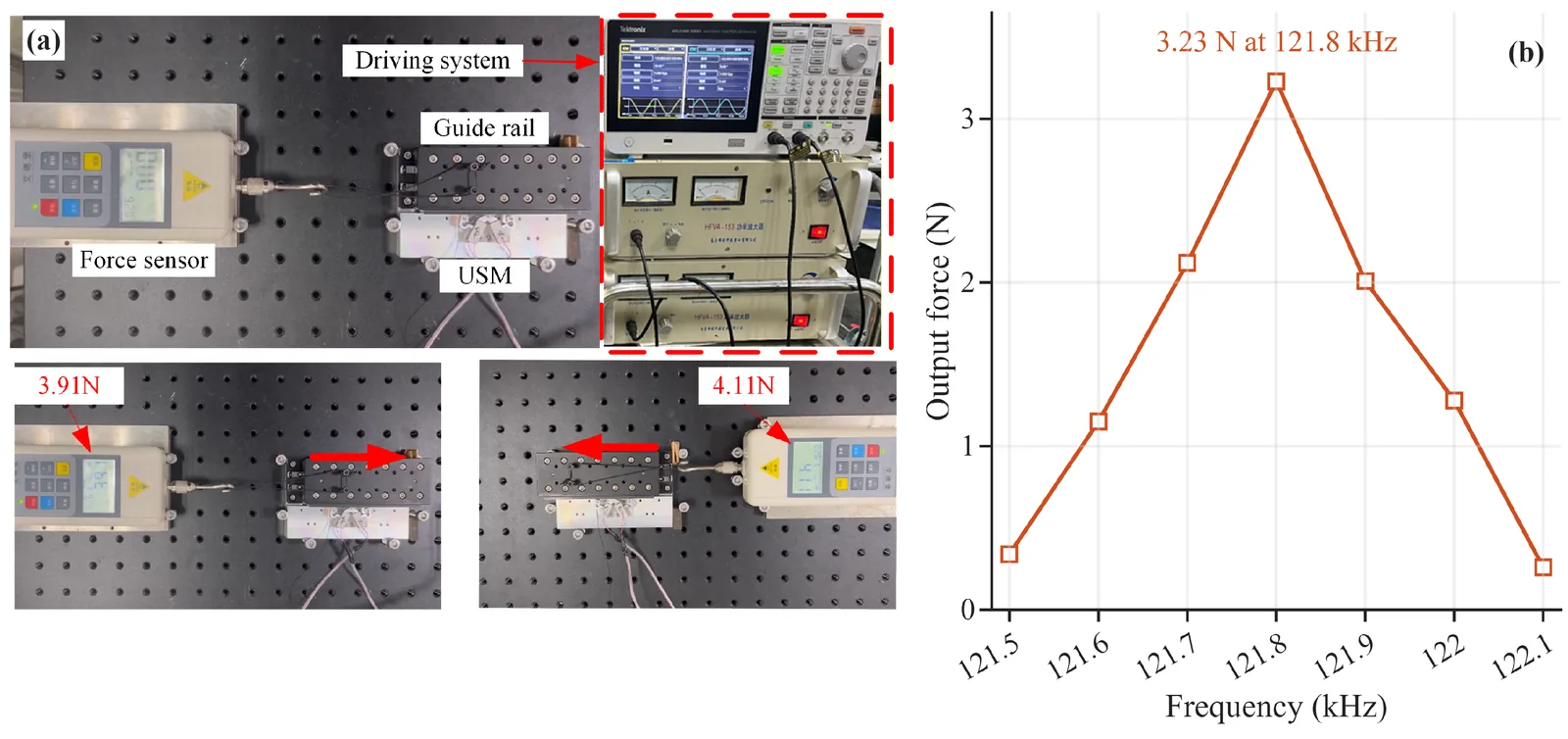
Load characteristics of the V-shaped USM: (**a**) bidirectional maximum-thrust test; (**b**) output force versus driving frequency.

The load-characterization system comprised an AFG3022 signal generator (Tektronix, Inc., Beaverton, OR, USA), an HFVP-153 power amplifier (Nanjing Foneng Technology Industrial Co., Ltd., Nanjing, China), a linear guide, a thin cord, and an HP-200 digital force gauge (Yueqing Aidebao Instrument Co., Ltd., Yueqing, China). The force gauge had a 0 N to 200 N measurement range and 0.01 N resolution. The drive frequency was first swept from 121.5 kHz to 122.1 kHz at 175 $V_{\mathrm{pp}}$. The output force increased from 0.34 N at 121.5 kHz to a maximum of 3.23 N at 121.8 kHz, and then decreased to 0.26 N at 122.1 kHz, as shown in Figure 12b. Therefore, 121.8 kHz was selected as the driving frequency for the subsequent maximum-thrust test. The voltage was then increased to 300 $V_{\mathrm{pp}}$. Reversing the phase sequence changed the motion direction, allowing the maximum thrust to be measured in both directions. Each direction was measured three times; the average rightward and leftward thrusts were 4.03 N and 3.87 N, respectively. As shown in Figure 12a, the maximum rightward and leftward thrusts were 4.11 N and 3.91 N, respectively, indicating approximately symmetric bidirectional output.

The thrust-to-mass ratio was defined as $\Gamma =F_{\max}/m_{s}$, where $F_{\max}$ is the maximum thrust and $m_{s}$ is the stator mass. Using the 4.11 N maximum thrust and 5.74 g stator mass in Table 10 gave a maximum ratio of 716 N/kg. This metric uses the stator mass and static maximum thrust; the base, actuating ring, and power electronics are not included in the mass denominator.

### 4.4. Microstepping Characteristics

**Figure 13:**
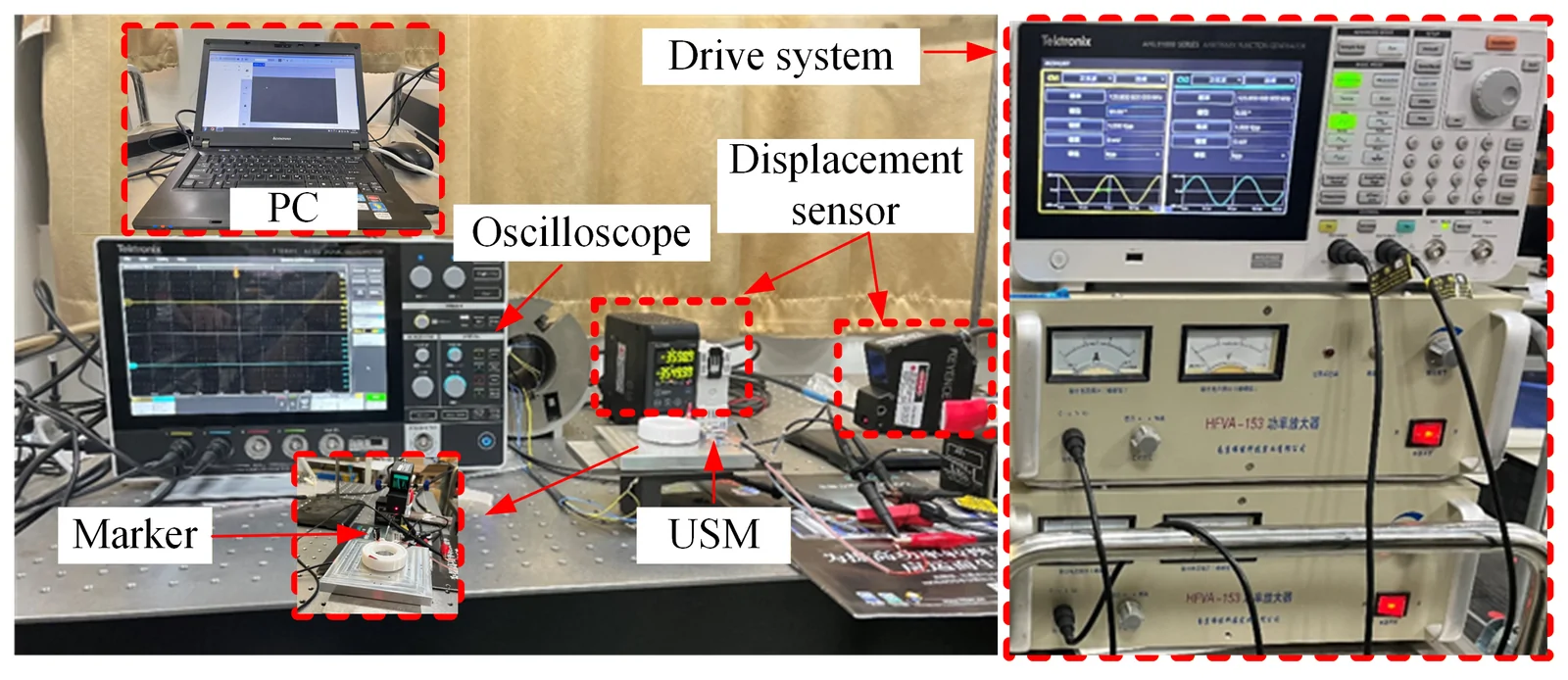
Test system for the microstepping characteristics of the USM.

For tangential microdisplacement measurement, a bearing-mounted test plate replaced the iris actuating ring. The system comprised the USM drive electronics, bearing test plate, oscilloscope, LK-H150 laser displacement sensor (KEYENCE Corporation, Osaka, Japan), and host computer, as shown in Figure 13. A marker bonded to the bearing surface allowed the small arc motion to be approximated as a tangential displacement. The number of excitation cycles was varied, and the displacement difference between adjacent stable plateaus was recorded.

**Figure 14:**
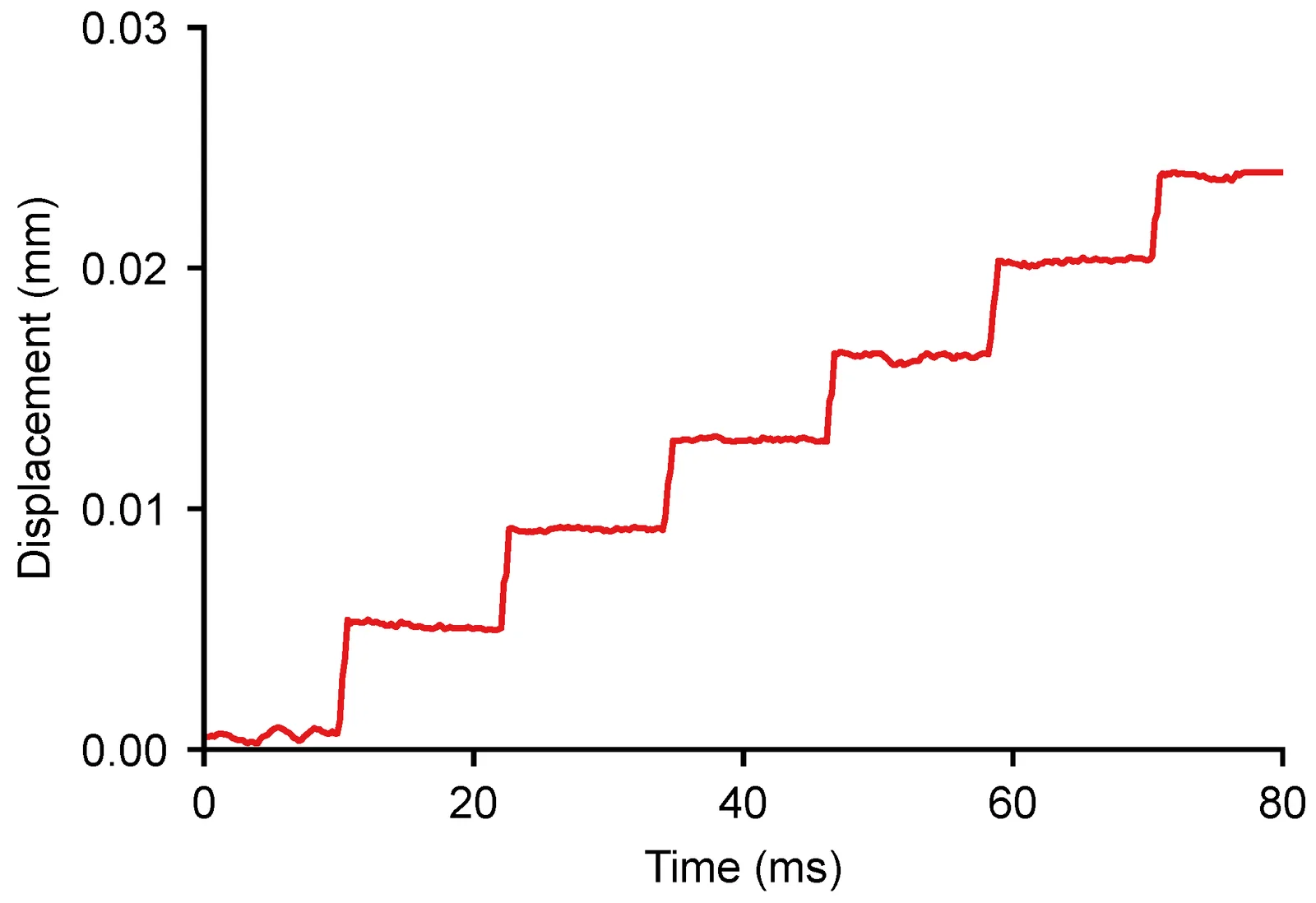
Measured stepwise displacement response under short-cycle excitation.

The measured response contained distinct, successive displacement plateaus, as shown in Figure 14. The minimum observed stable displacement difference was 3.23 μm, demonstrating micrometer-scale tangential stepping under this test configuration.

### 4.5. Temperature-Dependent Stator and Motor Characteristics

**Figure 15:**
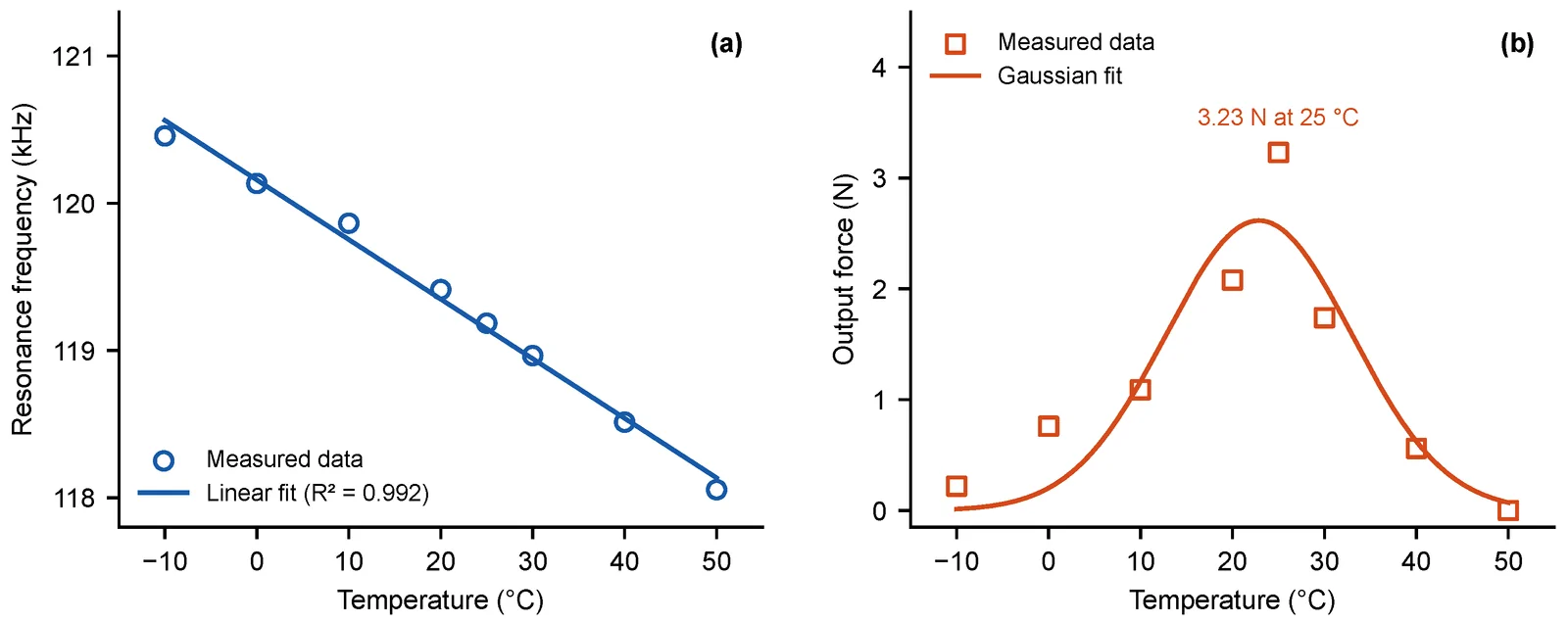
Temperature-dependent characteristics of the V-shaped USM: (**a**) resonance frequency and linear fit; (**b**) output force and single-Gaussian fit.

Two experiments were conducted at ambient pressure from −10 °C–50 °C to characterize actuator-level thermal sensitivity. The test stator was placed in a temperature chamber. The chamber temperature was varied from −10 °C to 50 °C at 2 °C/min. An impedance measurement was performed at each 10 °C temperature interval, with an additional measurement at the room-temperature point of 25 °C. After the chamber reached each set point, the stator was maintained at that temperature for 15 min to reach thermal equilibrium. One frequency sweep was then recorded using the PV520A impedance analyzer over 117 kHz to 122 kHz. The resonance frequency $f_{r}$ was extracted from each impedance spectrum. The measured resonance frequency decreased monotonically from 120.457 kHz at −10 °C to 118.054 kHz at 50 °C, corresponding to a total decrease of 2.403 kHz (1.99%), as shown in Figure 15a.

The V-shaped USM and an HP-200 digital force gauge were placed together in the temperature chamber. The chamber temperature was varied from −10 °C to 50 °C at 2 °C/min, and measurements were taken at 10 °C intervals, with an additional measurement at the room-temperature point of 25 °C. After each set point was reached, the motor and force gauge were maintained for 15 min before measurement. The motor was then driven at a fixed frequency of 121.8 kHz and a fixed voltage of 175 $V_{\mathrm{pp}}$. The output was blocked by the force gauge, and the output force $F_{\mathrm{out}}$ was recorded directly in newtons. A single measurement was recorded at each temperature condition (n=1). The output force increased from 0.22 N at −10 °C to a maximum of 3.23 N at 25 °C, and then decreased to 0 N at 50 °C, as shown in Figure 15b.

At 175 $V_{\mathrm{pp}}$, the frequency sweep in Figure 12b shows that the output force peaked at 121.8 kHz and decreased as the drive frequency moved away from this value. Figure 15a shows that the stator resonance shifted to lower frequencies as the temperature increased, while Figure 15b shows a corresponding force reduction under fixed 121.8 kHz excitation above 25 °C. These results indicate that thermally induced detuning contributed to the observed force reduction; temperature-dependent piezoelectric and contact properties may also have affected the response.

## 5. System Integration and Aperture-Regulation Performance

To evaluate direct actuation of the variable iris, the V-shaped USM was integrated with the actuating ring and 20 blades. An image-based aperture measurement and calibration method was first established. The effects of driving-cycle count, voltage, and frequency on the actuating-ring angle were then measured. Direction-specific cycle-to-angle gray-box models were identified for opening and closing, the local open-loop regulation error was evaluated, and quasi-static resistance and extended-aperture tests were used to characterize load variation and model extrapolation.

### 5.1. Integrated Test Platform and Aperture Measurement

**Figure 16:**
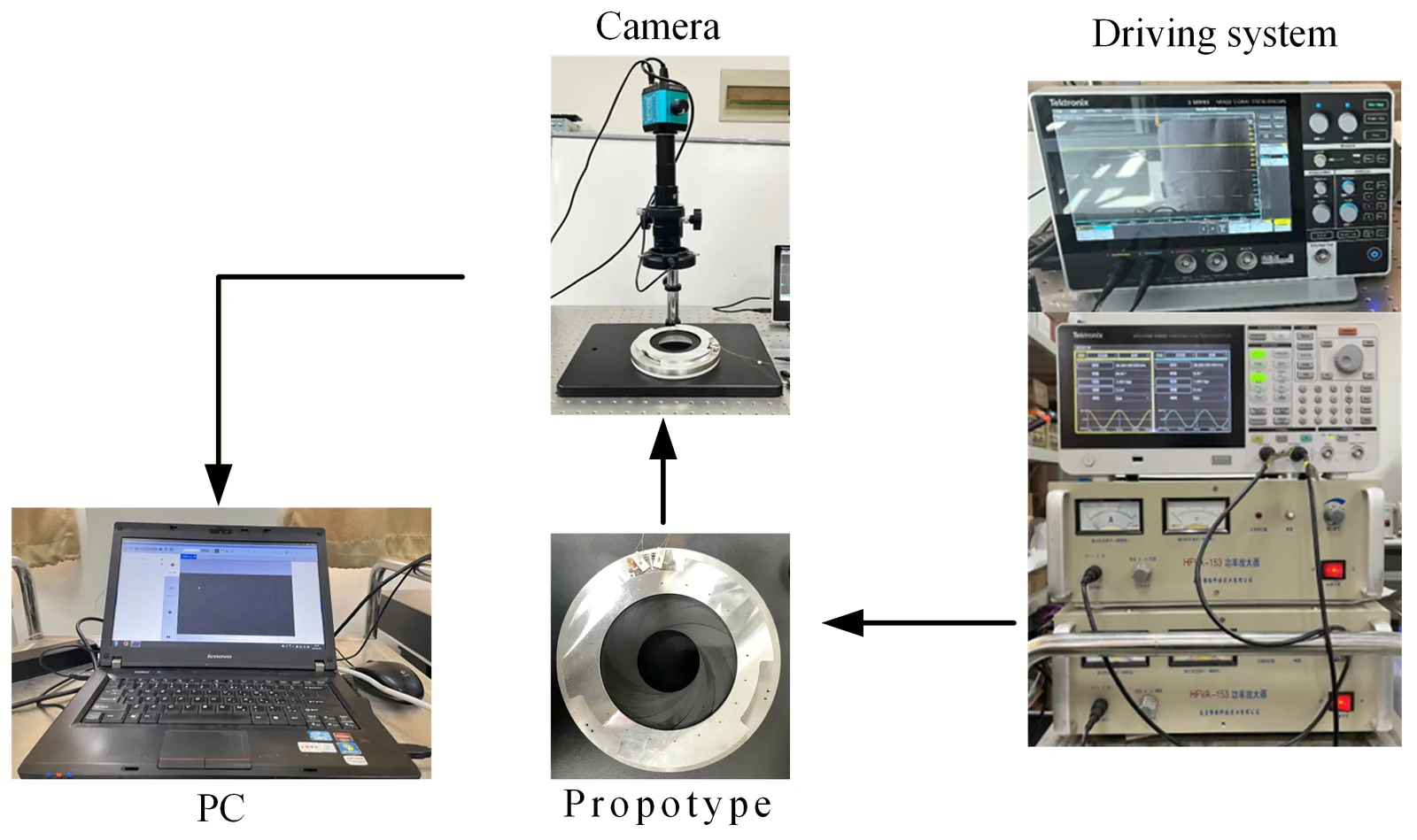
Experimental platform for the variable iris directly driven by the V-shaped USM.

The integrated platform comprised a signal generator, power amplifier, V-shaped USM, variable iris, P-103U digital microscope (Kunshan Gaopin Precision Instrument Co., Ltd., Kunshan, China), oscilloscope, and host computer, as shown in Figure 16. The microscope captured aperture images, while the host computer recorded the driving variables and converted the measured aperture into actuating-ring angle using the geometric mapping. Experiments were performed at room temperature over voltage, frequency, and cycle-count ranges of 50 $V_{\mathrm{pp}}$–200 $V_{\mathrm{pp}}$, 121.6 kHz–122.0 kHz, and 100–1500 cycles, respectively.

**Figure 17:**
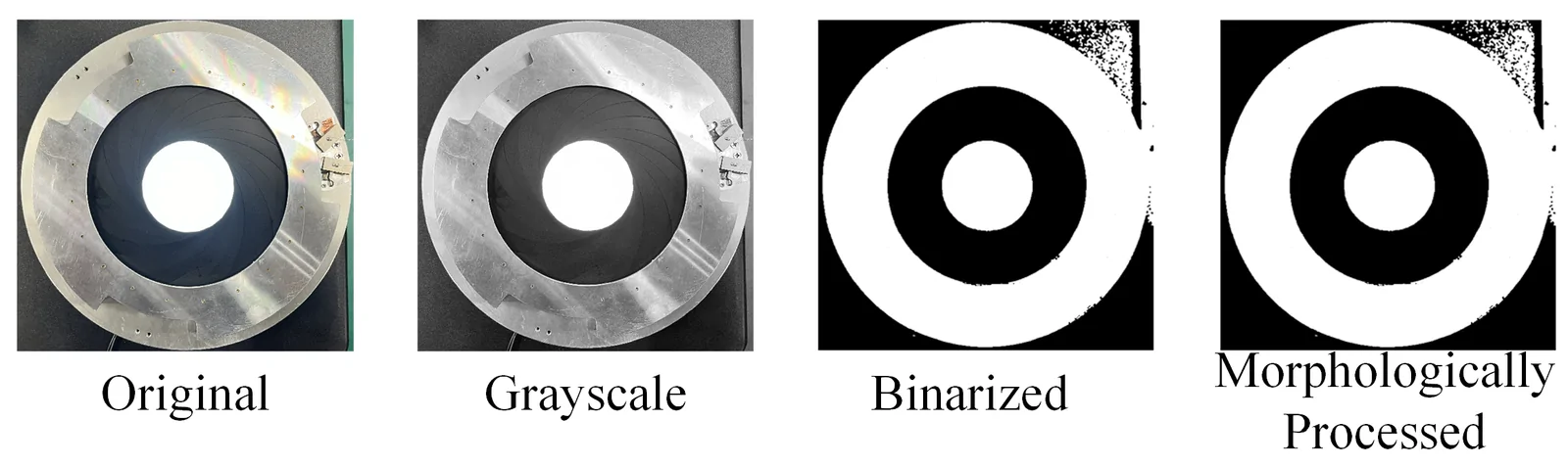
Grayscale conversion, binarization, and morphological processing of a variable-iris aperture image.

Each aperture image was converted to grayscale, binarized, and morphologically processed to suppress background noise and extract the inner aperture and outer reference contour, as shown in Figure 17. With the 98 mm-diameter outer contour being used as a calibration reference, the aperture diameter was calculated as

(9) $$D_{i}=D_{\mathrm{ref}}\frac{C_{i}}{C_{\mathrm{ref}}},$$

where $C_{i}$ and $C_{\mathrm{ref}}$ are the pixel perimeters of the measured aperture and reference contour, respectively. In the calibration image, $C_{i}=2089$ and $C_{\mathrm{ref}}=4706$, giving an aperture diameter of 43.5 mm. The calibrated diameter was then converted to actuating-ring angle through the geometric mapping.

### 5.2. Drive-Response Characterization and Direction-Specific Identification

**Figure 18:**
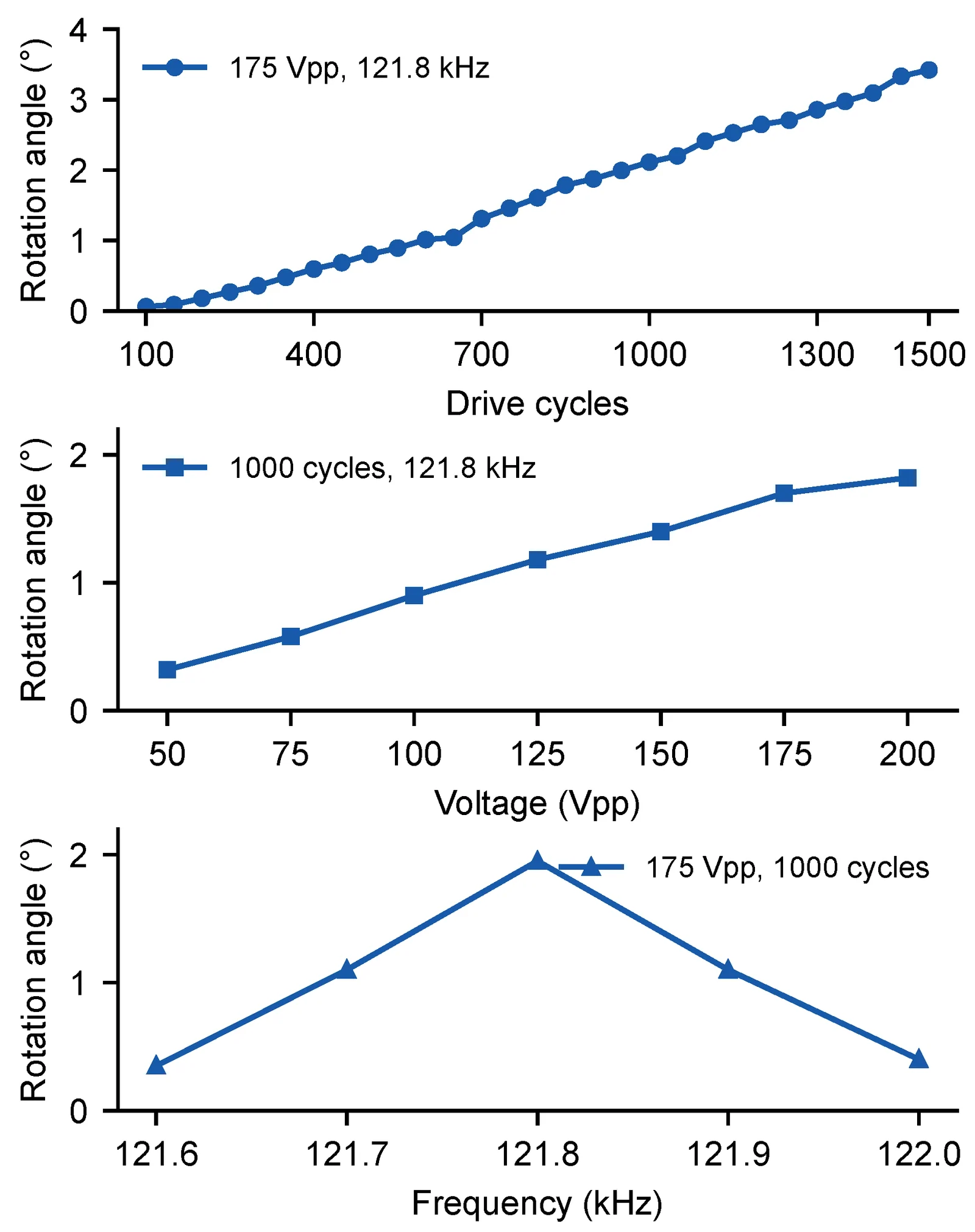
Measured relationships between actuating-ring rotation angle and driving-cycle count, voltage, and frequency.

Figure 18 shows the measured relationships between the actuating-ring angle and the three driving variables. At 175 $V_{\mathrm{pp}}$ and 121.8 kHz, the angle increased approximately linearly with the number of driving cycles, from approximately 0.06° at 100 cycles to approximately 3.42° at 1500 cycles. At 1000 cycles and 121.8 kHz, the angle also increased approximately linearly as the voltage increased from 50 $V_{\mathrm{pp}}$ to 200 $V_{\mathrm{pp}}$. However, changes in stator impedance with operating conditions made small voltage adjustments difficult to translate into stable angular increments.

At 175 $V_{\mathrm{pp}}$ and 1000 cycles, the angle–frequency response peaked near 121.8 kHz and exhibited a large angular change per unit frequency change. This steep response indicates potential sensitivity of fixed-frequency regulation to thermally induced resonance drift and vibration-related changes in contact conditions. Figure 18 characterizes this sensitivity under room-temperature conditions. In contrast, the cycle count is discrete, directly programmable, and monotonic over the tested range. The number of driving cycles was therefore selected as the input for the subsequent room-temperature identification and regulation tests.

Following the general gray-box parameter-estimation concept described by Kristensen et al. [33] and the direction-specific formulation of Lv et al. [31], the rotational relationship of the present actuating ring was prescribed as the deterministic model structure. The unknown parameters were estimated from the experimental data. The actuating ring was treated as a rigid rotating body, while blade friction, bearing resistance, and unmodeled loads were incorporated into equivalent parameters. Let $x_{1}\left(t\right)=\beta \left(t\right)$ be the actuating-ring angle, $x_{2}\left(t\right)=\dot{\beta }\left(t\right)$ the angular velocity, u(t) the normalized on–off pulse-train command to the USM, and y(t) the model output. During cycle-count identification, the carrier was fixed at 175 $V_{\mathrm{pp}}$ and 121.8 kHz; a command of *N* cycles corresponded to an excitation duration of $N/f_{d}$, where $f_{d}$ is the carrier frequency. The simplified state model is

(10) $$\begin{array}{cc} {\dot{x}}_{1}\left(t\right) & =x_{2}\left(t\right),\\ {\dot{x}}_{2}\left(t\right) & =-gx_{2}^{2}\left(t\right)+px_{2}\left(t\right)+bu\left(t\right)+q,\\ y(t) & =x_{1}\left(t\right). \end{array}$$

The signed equivalent parameters *g*, *p*, *b*, and *q* were estimated from the measurements. Because friction direction and mechanism backlash can produce different opening and closing responses, the two directions were identified separately. The coefficients are effective parameters of the normalized input–output model and are not interpreted as independently measured physical constants. With $s={\left[g,p,b,q\right]}^{T}$, nonlinear least squares minimized the squared difference between model output and experiment,

(11) $$J\left(s\right)=\sum_{i=1}^{n}{\left[y_{m}\left(t_{i};s\right)-y_{e}\left(t_{i}\right)\right]}^{2},$$

where $y_{m}$ and $y_{e}$ are the model output and measured response. The parameters were iteratively identified using the Levenberg–Marquardt algorithm, and the results are listed in Table 11. Model agreement was quantified using the normalized root-mean-square fit returned by the System Identification Toolbox in MATLAB R2025b (The MathWorks, Inc., Natick, MA, USA),

(12) $$\mathrm{Fit}=100\left(1-\frac{{∥y_{e}-y_{m}∥}_{2}}{{∥y_{e}-{\bar{y}}_{e}∥}_{2}}\right)\%.$$

**Figure 19:**
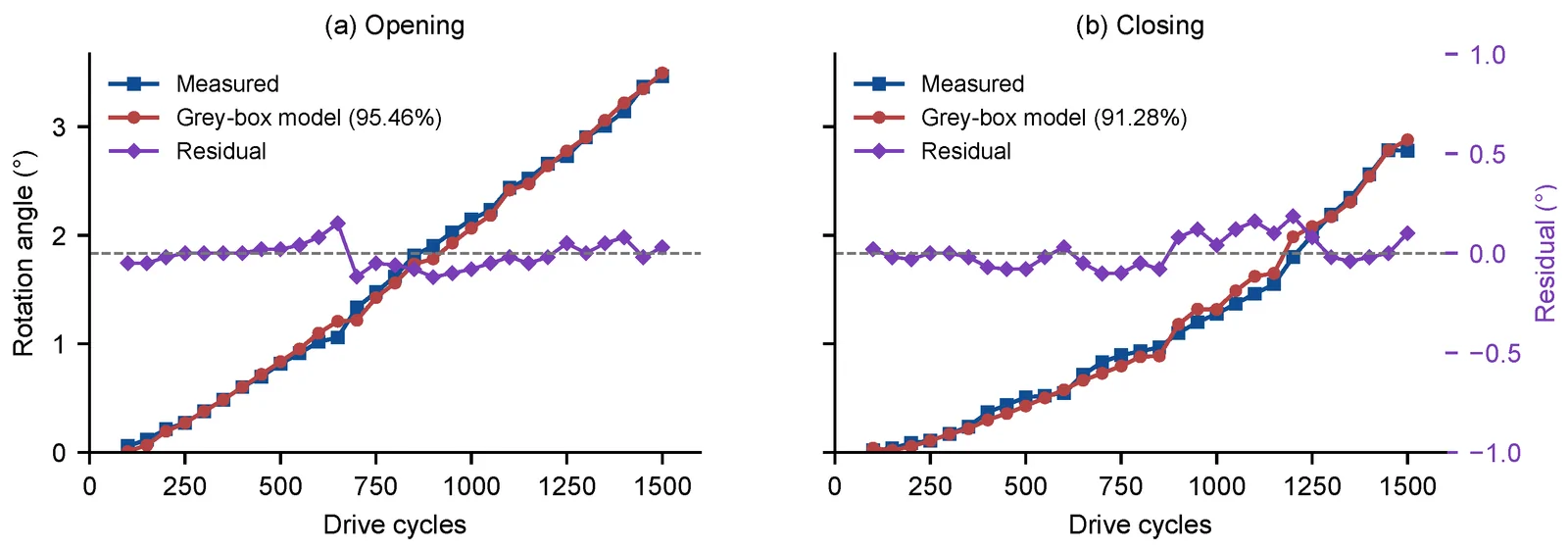
Grey-box identification results and residuals: (**a**) opening; and (**b**) closing. The grey dashed line indicates zero residual.

For opening, the model followed the measured trend with a fit of 95.46%, as shown in Figure 19a. The closing fit was 91.28% (Figure 19b). The lower closing fit is consistent with the combined effects of blade compression, reversed friction direction, and mechanism backlash.

**Figure 20:**
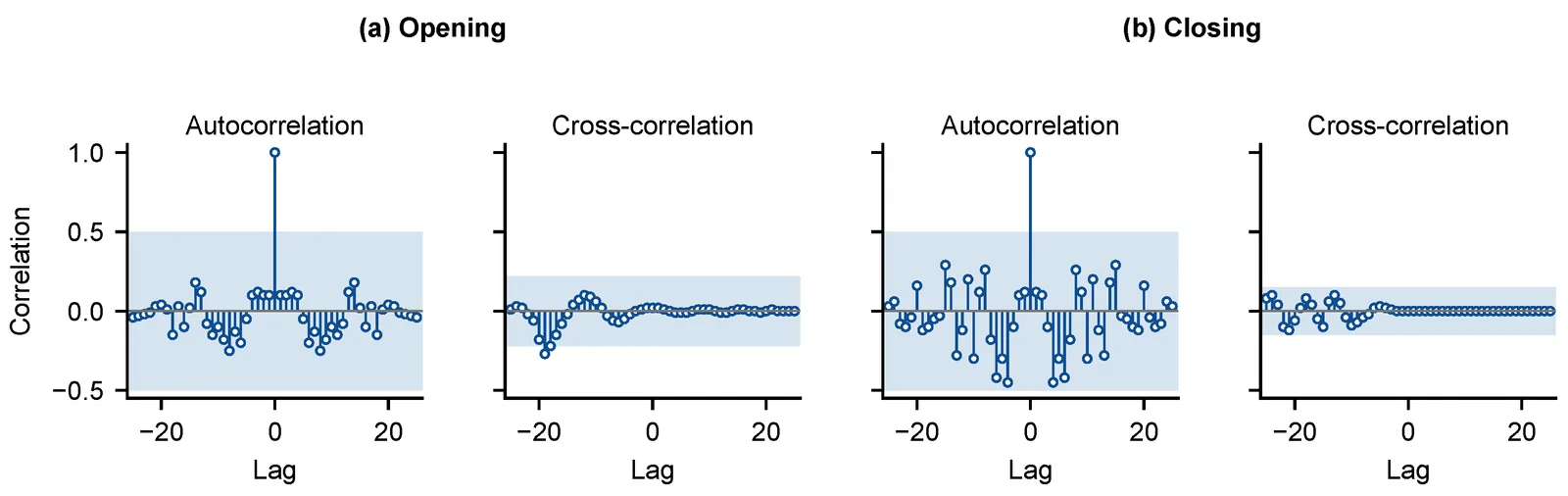
Residual correlation analysis of the gray-box models: (**a**) opening; and (**b**) closing. The blue shaded bands indicate the confidence bounds generated by the residual-analysis function, and the open circles and stems show the calculated correlation coefficients.

Residual autocorrelation and input–residual cross-correlation were further examined for opening and closing, as shown in Figure 20a,b. The shaded regions are the 99% confidence bounds generated by the MATLAB residual-analysis function. Except for the necessary autocorrelation peak at zero lag, the coefficients generally remained within the bounds, and the cross-correlation did not show a persistent systematic trend. The models therefore captured the dominant input–output relationships under the tested drive range and sampling conditions.

### 5.3. Open-Loop Regulation over a Local Aperture Range

Thirteen open-loop regulation tests, which were not used to estimate the model coefficients, were conducted over a 10 mm–20 mm aperture range at room temperature, 175 $V_{\mathrm{pp}}$, and 121.8 kHz. For each target aperture, the corresponding reference ring angle $\beta_{r,i}$ was determined, the required driving-cycle count was calculated from the identified model, and the actual ring angle $\beta_{e,i}$ was obtained by image measurement. The angular error and mean absolute error (MAE) were defined as

(13) $$e_{i}=\beta_{e,i}-\beta_{r,i},\;\mathrm{MAE}=\frac{1}{n}\sum_{i=1}^{n}\left|e_{i}\right|.$$

**Figure 21:**
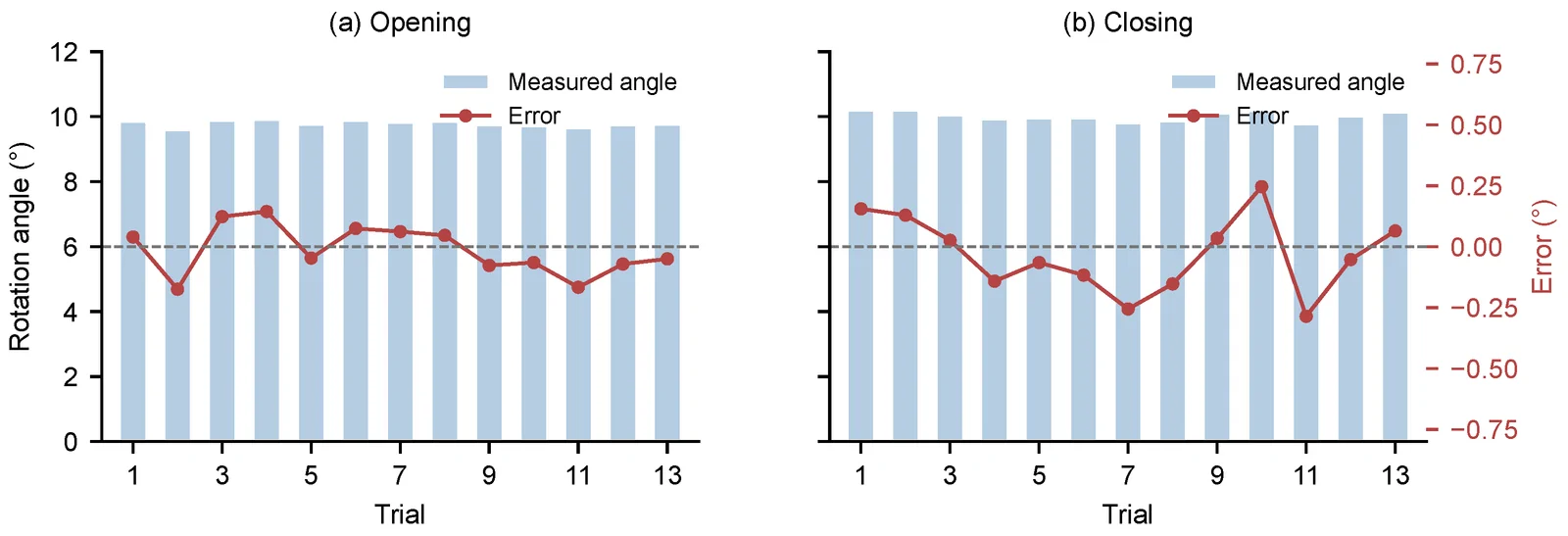
Measured actuating-ring angles and open-loop regulation errors: (**a**) aperture opening; and (**b**) aperture closing. The grey dashed line denotes zero angular error.

Figure 21a,b shows the measured ring angles and regulation errors during opening and closing. The opening errors ranged from −0.175° to 0.144°, while the closing errors ranged from −0.286° to 0.246°.

The opening and closing MAEs were 0.087° and 0.13°, respectively. Over the 10 mm–20 mm aperture interval, the total actuating-ring travel was approximately 9.9°; the maximum absolute error of 0.286° corresponded to approximately 2.9% of this travel. The larger closing error is consistent with the combined influence of blade compression, friction direction, and aperture-boundary irregularity on the mechanism response. The local regulation results are summarized in Table 12.

These local tests quantified actuating-ring-angle regulation over the 10 mm–20 mm room-temperature interval and verified bidirectional motion of the actuating ring and 20 blades.

### 5.4. Full-Range Driving Resistance and Model-Extrapolation Validation

**Figure 22:**
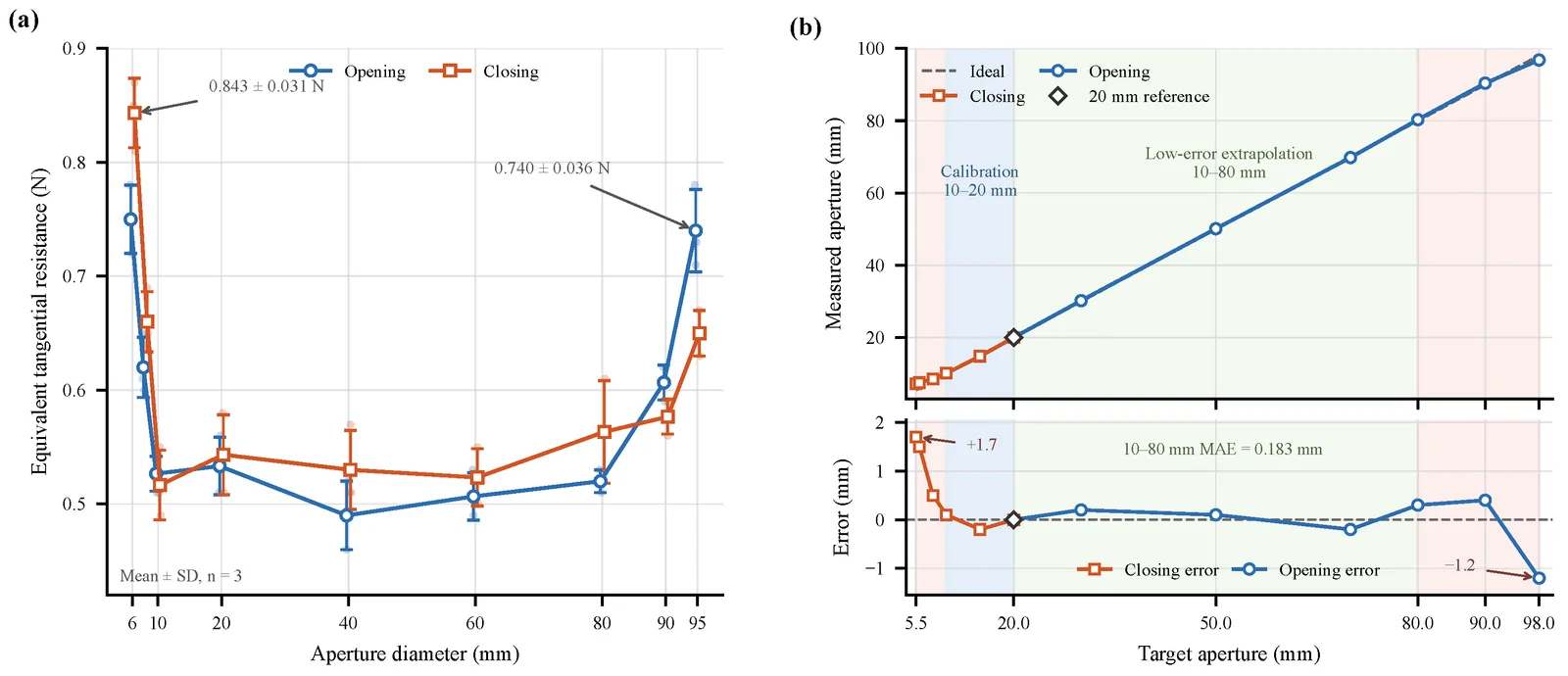
Full-range performance of the variable iris: (**a**) aperture-dependent quasi-static equivalent tangential resistance; (**b**) full-range model-extrapolation validation from a 20 mm reference. In panel (**b**), the red shaded regions indicate the two near-limit aperture intervals ( 5.5 mm–10 mm and 80 mm–98 mm), while the grey dashed lines denote the ideal measured-equals-target relationship and zero aperture error.

To evaluate the variation in driving load caused by aperture-dependent blade overlap, the quasi-static driving resistance was measured in the opening and closing directions at aperture diameters of 6, 8, 10, 20, 40, 60, 80, 90, and 95 mm. A slowly varying tangential load was applied to the actuating ring, and the measured load was converted to an equivalent tangential resistance at the driving-tip contact position using the moment-arm relationship. Three measurements were recorded for each aperture and direction; the means and sample standard deviations are shown in Figure 22a. At 6 mm, the closing and opening resistances were 0.843±0.031 N and 0.750±0.030 N, respectively. From 10 mm to 80 mm, the directional means were mainly distributed between 0.490 N and 0.563 N. At 95 mm, the opening and closing resistances increased to 0.740±0.036 N and 0.650±0.020 N, respectively. The driving resistance increased near both ends of the aperture range and remained lower over the intermediate range. The 3.23 N peak output force measured at 175 $V_{\mathrm{pp}}$ in Figure 12b was approximately 3.8 times the maximum mean quasi-static resistance, indicating a tangential force margin under the measured quasi-static load conditions.

To evaluate extrapolation of the model identified over the local 10 mm–20 mm interval, 20 mm was used as a common reference aperture, and the identified model was used to calculate the driving-cycle counts for extended target apertures. Closing tests were performed from the reference toward 5.5 mm, and opening tests were performed from the reference toward 98 mm. The resulting aperture was measured by image processing. The aperture error was defined as

(14) $$e_{D,i}=D_{e,i}-D_{r,i},$$

where $D_{r,i}$ and $D_{e,i}$ are the target and measured apertures, respectively. One measurement was recorded at each target aperture, as shown in Figure 22b. Excluding the 20 mm reference point, the six validation points from 10 mm to 80 mm yielded an aperture MAE of 0.183 mm. For a target of 5.5 mm, the measured aperture was 7.2 mm, giving an error of 1.7 mm; for a target of 98 mm, the measured aperture was 96.8 mm, giving an error of −1.2 mm. Thus, the locally identified model retained a small extrapolation error over the extended 10 mm–80 mm range, whereas the errors increased near the minimum and maximum apertures. This trend is consistent with the higher quasi-static resistance measured near both ends in Figure 22a.

The numerical data corresponding to the manuscript tables and data-bearing figures are provided in Data S1.

## 6. Conclusions

This study established a peripheral direct-drive architecture for a 20-blade variable iris. The V-shaped mode-coupled USM acts directly on the actuating-ring edge, avoiding the separate motor rotor and connecting ring used in the earlier arrangement. A wave-spring–bearing load path accommodates blade-stack displacement while permitting circumferential ring rotation. The mechanism was geometrically designed for a 5.5 mm–98 mm aperture range, and FEA supported the intended stiffness allocation between the blade stack and compliant preload mechanism.

The two-stage stator procedure separated unclamped frequency matching from optimization under the actual flexible clamping boundary. The final model predicted a 170 Hz working-mode separation and a 14.83 kHz minimum gap to adjacent modes. The measured working modes were separated by 800 Hz. This larger experimental separation indicates sensitivity to adhesive layers, geometric tolerances, and the realized clamping boundary, although both measured modes remained within the intended operating band. Electrical measurements showed closely clustered characteristics among the tested stators, while vibration, thrust, and short-cycle tests demonstrated bidirectional thrust up to 4.11 N and micrometer-scale tangential stepping. From −10 °C to 50 °C, the measured resonance frequency decreased by 2.403 kHz; the output force reached 3.23 N at 25 °C and decreased to 0 N at 50 °C. Together, these measurements verified the operating behavior of the optimized stator under the tested drive and temperature conditions.

Integration tests confirmed bidirectional motion of the actuating ring and 20 blades at room temperature. Quantitative regulation over 10 mm–20 mm produced mean absolute ring-angle errors of 0.087° during opening and 0.13° during closing. The maximum mean quasi-static equivalent tangential resistance was 0.843 N, while the peak motor output at 121.8 kHz and 175 $V_{\mathrm{pp}}$ was 3.23 N. Extrapolation tests from a 20 mm reference produced an aperture MAE of 0.183 mm over 10 mm–80 mm; the errors were 1.7 mm and −1.2 mm at the 5.5 mm and 98 mm targets, respectively, showing increased prediction error near the travel limits. The study focuses on structural design and direct-drive validation, while the temperature tests characterize actuator-level thermal response. Future work will address the coupled nonlinear effects of resonance drift, piezoelectric response, and frictional contact, together with temperature compensation, frequency tracking, paired full-stroke bidirectional hysteresis, retention, and environmental testing.

## Figures and Tables

**Table 1:** Principal design requirements and prototype parameters of the variable iris.

Item	Design or Implemented Value	Implication for Actuator Design
Designed clear-aperture range	5.5 mm–98 mm	Large actuating-ring rotation and continuous regulation
Local model-identification and bidirectional-regulation interval	10 mm–20 mm	Local room-temperature open-loop regulation
Model-extrapolation validation targets	5.5 mm–98 mm	Extended validation from a 20 mm reference
Number of blades	20	Aperture-dependent friction generated by blade overlap
Overall envelope	170 mm × 170 mm × 33.5 mm	Restricted axial transmission and motor installation space
Drive type	Direct friction drive at the outer ring edge	High bidirectional thrust and potential self-holding behavior
Regulation capability	Continuous aperture regulation and motor microstepping	Stable working modes and micrometer-scale motor steps

**Table 2:** Geometric and material parameters of the compliant preload mechanism.

Parameter	Symbol	Value	Unit
Number of waves	$N_{w}$	4	–
Inner diameter	$b_{1}$	107.5	mm
Outer diameter	$b_{2}$	112.6	mm
Mean diameter	$b_{4}$	110.05	mm
Free height	$h_{0}$	7	mm
Band width	$b_{3}$	2.5	mm
Thickness	$t_{0}$	1.5	mm
Material	–	Polytetrafluoroethylene	–
Young’s modulus	$E_{1}$	0.28	GPa
Poisson’s ratio	ν	0.4	–
Target axial load	–	3	N
Equivalent axial stiffness (FEA)	$k_{p}$	2.96	N/mm

**Table 3:** Finite-element stiffness comparison between the preload mechanism and three overlapping blades.

Object	Axial Load (N)	Maximum Deformation (mm)	Equivalent Stiffness (N/mm)
Preload mechanism	3	1.0081	2.96
Three overlapping blades	3	0.13387	22.4

**Table 4:** Control factors and levels for the Taguchi design of the unclamped stator (unit: mm).

Factor	Level 1	Level 2	Level 3	Level 4	Level 5
$L_{1}$	1.9	2.0	2.1	2.2	2.3
$L_{2}$	15.1	15.2	15.3	15.4	15.5
$R_{1}$	9.3	9.4	9.5	9.6	9.7
$R_{2}$	1.2	1.3	1.4	1.5	1.6
*T*	3.2	3.3	3.4	3.5	3.6

**Table 5:** $L_{25}\left(5^{5}\right)$ orthogonal array and confirmation results for the unclamped stator.

Run	$L_{1}$ (mm)	$L_{2}$ (mm)	$R_{1}$ (mm)	$R_{2}$ (mm)	*T* (mm)	Δf (Hz)
1	1.9	15.1	9.3	1.2	3.2	2160
2	1.9	15.2	9.4	1.3	3.3	1660
3	1.9	15.3	9.5	1.4	3.4	350
4	1.9	15.4	9.6	1.5	3.5	370
5	1.9	15.5	9.7	1.6	3.6	840
6	2.0	15.1	9.4	1.4	3.5	970
7	2.0	15.2	9.5	1.5	3.6	440
8	2.0	15.3	9.6	1.6	3.2	720
9	2.0	15.4	9.7	1.2	3.3	1110
10	2.0	15.5	9.3	1.3	3.4	1180
11	2.1	15.1	9.5	1.6	3.3	360
12	2.1	15.2	9.6	1.2	3.4	960
13	2.1	15.3	9.7	1.3	3.5	470
14	2.1	15.4	9.3	1.4	3.6	470
15	2.1	15.5	9.4	1.5	3.2	380
16	2.2	15.1	9.6	1.3	3.6	390
17	2.2	15.2	9.7	1.4	3.2	350
18	2.2	15.3	9.3	1.5	3.3	1010
19	2.2	15.4	9.4	1.6	3.4	640
20	2.2	15.5	9.5	1.2	3.5	1340
21	2.3	15.1	9.7	1.5	3.4	750
22	2.3	15.2	9.3	1.6	3.5	820
23	2.3	15.3	9.4	1.2	3.6	1300
24	2.3	15.4	9.5	1.3	3.2	730
25	2.3	15.5	9.6	1.4	3.3	380
Confirmation	2.1	15.3	9.5	1.5	3.3	310

**Table 6:** Design variables and predicted driving-tip amplitudes of the three optimization candidates.

Candidate	$d_{1}$ (mm)	$d_{2}$ (mm)	$d_{3}$ (mm)	$d_{4}$ (mm)	$d_{5}$ (°)	Tangential Amplitude (μm)	Normal Amplitude (μm)
1	2.8	3.2	1.3	1.4	135.7	2881.4	1064.5
2	2.9	3.7	1.4	1.5	135.9	1620.8	982.6
3	3.2	3.7	1.4	1.4	135.8	705.8	576.7

**Table 7:** Modal verification of the three flexible-clamping candidates.

Candidate	Adjacent Mode 1 (kHz)	Working Mode 1 (kHz)	Working Mode 2 (kHz)	Adjacent Mode 2 (kHz)	Minimum Separation (kHz)
1	118.90	121.96	122.60	142.77	3.06
2	103.53	121.96	122.21	133.90	11.69
3	105.96	121.93	122.10	136.93	14.83

**Table 8:** Statistical ranges of the electrical characteristics of ten stators.

Metric	Phase-A Range	Phase-B Range	Principal Observation
Static capacitance (nF)	2.113–2.209	2.120–2.286	Concentrated near 2.2 nF
Series-resonance frequency (Hz)	118,972–120,098	118,990–120,233	Similar between phases
Parallel-resonance frequency (Hz)	120,060–122,240	119,960–122,240	Larger deviations in individual samples

**Table 9:** Comparison of simulated and measured working-mode frequencies.

Source	Symmetric Mode (kHz)	Antisymmetric Mode (kHz)	Mode Separation (Hz)
Finite-element analysis	122.10	121.93	170
Experiment	121.50	120.70	800
Relative frequency deviation	0.5%	1.0%	–

**Table 10:** Load performance of the V-shaped USM.

Stator Mass (g)	Maximum Rightward Thrust (N)	Maximum Leftward Thrust (N)	Average Rightward Thrust (N)	Average Leftward Thrust (N)	Maximum Thrust-to-Mass Ratio (N/kg)
5.74	4.11	3.91	4.03	3.87	716

**Table 11:** Identified parameters of the direction-specific grey-box models.

Direction	*g*	*p*	*b*	*q*
Opening	70.38	111.52	0.0200	−43.14
Closing	−6.19	−15.85	0.0035	6.67

**Table 12:** Open-loop regulation performance over the tested local aperture range.

Direction	Model Fit (%)	Angular-Error Range (°)	MAE (°)
Opening	95.46	−0.175–0.144	0.087
Closing	91.28	−0.286–0.246	0.13

## Data Availability

The original contributions presented in this study are included in the article and its Supplementary Materials. Further inquiries can be directed to the corresponding authors.

## Supplementary Materials

The following supporting information can be downloaded at https://www.mdpi.com/article/10.3390/mi17091089/s1.

## References

[1] Tian Q., Chang S., He F., Li Z., Qiao Y. (2017). Spherical Warm Shield Design for Infrared Imaging Systems. Infrared Phys. Technol..

[2] Gat N., Zhang J., Li M.D., Chen L., Gurrola H. (2007). Variable Cold Stop for Matching IR Cameras to Multiple f-number Optics. Proceedings of SPIE.

[3] Kim W.S., Lee D.J., Lee S.K. (2011). Influence of a High Vacuum on the Precise Positioning Using an Ultrasonic Linear Motor. Rev. Sci. Instrum..

[4] Morita T., Niino T., Asama H. (2002). Rotational Feedthrough Using Ultrasonic Motor for High Vacuum Condition. Vacuum.

[5] Liu X., Zhao G., Qiu J. (2021). Improving the Performance of Ultrasonic Motors in Low-Pressure, Variable-Temperature Environments. Tribol. Int..

[6] Seo H.W., Chae J.B., Hong S.J., Rhee K., Chang J.H., Chung S.K. (2015). Electromagnetically Driven Liquid Iris. Sens. Actuators A Phys..

[7] Lee J., Park Y., Chung S.K. (2019). Multifunctional Liquid Lens for Variable Focus and Aperture. Sens. Actuators A Phys..

[8] Abuseada M., Ozalp N. (2020). Experimental and Numerical Study on a Novel Energy Efficient Variable Aperture Mechanism for a Solar Receiver. Sol. Energy.

[9] Li X., Zhou S. (2016). A Novel Piezoelectric Actuator with a Screw-Coupled Stator and Rotor for Driving an Aperture. Smart Mater. Struct..

[10] Gao X., Yang J., Wu J., Xin X., Li Z., Yuan X., Shen X., Dong S. (2020). Piezoelectric Actuators and Motors: Materials, Designs, and Applications. Adv. Mater. Technol..

[11] Zhou X., Wu S., Wang X., Wang Z., Zhu Q., Sun J., Huang P., Wang X., Huang W., Lu Q. (2024). Review on Piezoelectric Actuators: Materials, Classifications, Applications, and Recent Trends. Front. Mech. Eng..

[12] Ryndzionek R., Sienkiewicz L. (2021). A Review of Recent Advances in the Single- and Multi-Degree-of-Freedom Ultrasonic Piezoelectric Motors. Ultrasonics.

[13] Naz S., Xu T.B. (2024). A Comprehensive Review of Piezoelectric Ultrasonic Motors: Classifications, Characterization, Fabrication, Applications, and Future Challenges. Micromachines.

[14] Li J., Wang Y., Chen Z., Cheng F., Yu Q. (2021). A Compact Linear Ultrasonic Motor Composed by Double Flexural Vibrator. Micromachines.

[15] Li Z., Yi X., Zhu R., Yu Z., Yuan X., PourhosseiniAsl M., Dong S. (2023). A Symmetric-Actuating Linear Piezoceramic Ultrasonic Motor Capable of Producing a Scissoring Effect. Research.

[16] Liu Z., Fu Q., Yang P., Dong Z., Zhang L., Yao Z. (2023). Design and Performance Evaluation of a Miniature I-Shaped Linear Ultrasonic Motor with Two Vibrators. Ultrasonics.

[17] Kikuchi K., Hussain M., Mashimo T. (2023). Fabrication and Characterization of a Submillimeter-Scale Ultrasonic Motor. Sens. Actuators A Phys..

[18] Ryndzionek R., Sienkiewicz L., Michna M., Chodnicki M. (2021). Design Evolution of the Ultrasonic Piezoelectric Motor Using Three Rotating Mode Actuators. IEEE Access.

[19] Liu J., Gao X., Jin H., Ren K., Guo J., Qiao L., Qiu C., Chen W., He Y., Dong S. (2022). Miniaturized Electromechanical Devices with Multi-Vibration Modes Achieved by Orderly Stacked Structure with Piezoelectric Strain Units. Nat. Commun..

[20] Dong Z., Xu L. (2023). Performance Analysis and Experimental Research of a Dual-Vibrator Traveling Wave Ultrasonic Motor. Micromachines.

[21] Pan Z., Wang L., Yang Y., Jin J., Qiu J. (2023). A Novel Bonded-Type 3-Degree-of-Freedom Ultrasonic Motor: Design, Simulation, and Experimental Investigation. Smart Mater. Struct..

[22] Izuhara S., Mashimo T. (2023). Tilting Ultrasonic Motor Using Extension and Shear Vibration Modes. Sens. Actuators A Phys..

[23] Ma X., Yang Y., Qiu J., Zhang J., Vasiljev P., Wu J., Mazeika D., Zhao L., Borodinas S., Liu J. (2024). A Novel Rotary Ultrasonic Motor Based on Multiple Langevin Transducers: Design, Simulation, and Experimental Investigation. Smart Mater. Struct..

[24] Jian Y., Liu Z., Yao P., Zhou W., He J., Liang H. (2025). A Novel Motion Platform Based on Dual Driving Feet Linear Ultrasonic Motor. Micromachines.

[25] Wang L., Wang X., Jin J., Yu P., Luo G. (2021). Transfer Matrix Modeling on a Longitudinal-Bending Coupled Piezoelectric Transducer with Single-Phase Excitation: Analysis and Verification. Rev. Sci. Instrum..

[26] Lu D., Liu H., Xu J., Yang T., Hu H. (2022). A Linear Ultrasonic Motor Based on Coupling Vibration Mode. Micromachines.

[27] Qu H., Liu C., Zhang L., Qu J., Song B. (2022). A Longitudinal-Bending Hybrid Linear Ultrasonic Motor and Its Driving Characteristic. Shock Vib..

[28] Cao T., Li X., Wang B., Mi Y., Zhao G., Twiefel J., Wu D. (2020). Viscoelastic Analytical Model and Design of Polymer-Based Bimodal Piezoelectric Motor. Mech. Syst. Signal Process..

[29] Jian Y., Liu Z., He J., Zhou W., Liang H. (2024). Development of a Plate Linear Ultrasonic Motor Using the Power Flow Method. Micromachines.

[30] Geng R., Yao Z., Wang Y., Huang J., Liu H. (2022). Analysis and Optimization of a Microgripper Driven by Linear Ultrasonic Motors. Micromachines.

[31] Lv B., Li X., Wu J., Tang L., Yang F., Sun H., Gan Y., Li S., Li M. (2024). Ultrasonic Motor-Driven Variable Aperture Open-Loop Control Method. Infrared Laser Eng..

[32] Dragoni E. (1988). A Contribution to Wave Spring Design. J. Strain Anal. Eng. Des..

[33] Kristensen N.R., Madsen H., Jørgensen S.B. (2004). Parameter Estimation in Stochastic Grey-Box Models. Automatica.

