# Supplementary material for: A Large-Aperture Variable Iris Directly Driven by a V-Shaped Mode-Coupled Ultrasonic Motor: Design, Optimization, and Experimental Validation
Source: Micromachines (Basel). 2026 Sep 16;17(9):1089. doi: 10.3390/mi17091089 (PMC13609041; doi:10.3390/mi17091089)
Supplement: Supplementary file 1 [file micromachines-17-01089-s001.zip › micromachines-4520537-supplementary.pdf]

Data S1: Minimal Dataset for Manuscript Tables and Data-Bearing Figures

A Large-Aperture Variable Iris Directly Driven by a V-Shaped Mode-Coupled Ultrasonic Motor

Benchao Lv et al.

This document compiles the numerical data corresponding to the tables and data-bearing figures in the accepted manuscript. Manuscript table and figure numbers follow the current production proof for manuscript micromachines-4520537.

Contents

1 Numerical Data Corresponding to Manuscript Tables **2**

2 Point-by-Point Data for Data-Bearing Figures **4**

2.1 Manuscript Figure 14: Stepwise Displacement Response 4

2.2 Manuscript Figure 15: Temperature-Dependent Characteristics 11

2.3 Manuscript Figure 18: Drive-Response Characterization 11

2.4 Manuscript Figure 19: Direction-Specific Identification 12

2.5 Manuscript Figure 20: Residual Correlation Analysis 13

2.6 Manuscript Figure 21: Open-Loop Regulation Results 15

2.7 Manuscript Figure 22: Full-Range Resistance and Model Extrapolation 16

3 Key Numerical Results **17**

4 File and Variable Guide **17**

1 Numerical Data Corresponding to Manuscript Tables

Table S1: Data corresponding to Manuscript Table 1: principal design requirements and prototype parameters of the variable iris.

Item	Design or implemented value	Implication for actuator design
Designed clear-aperture range	5.5–98 mm	Large actuating-ring rotation and continuous regulation
Local model-identification and bidirectional-regulation interval	10–20 mm	Local room-temperature open-loop regulation
Model-extrapolation validation targets	5.5–98 mm	Extended validation from a 20 mm reference
Number of blades	20	Aperture-dependent friction generated by blade overlap
Overall envelope	170 mm × 170 mm × 33.5 mm	Restricted axial transmission and motor installation space
Drive type	Direct friction drive at the outer ring edge	High bidirectional thrust and potential self-holding behavior
Regulation capability	Continuous aperture regulation and motor microstepping	Stable working modes and micrometer-scale motor steps

Table S2: Data corresponding to Manuscript Table 2: geometric and material parameters of the compliant preload mechanism.

Parameter	Symbol	Value	Unit
Number of waves	N_w	4	–
Inner diameter	b_1	107.5	mm
Outer diameter	b_2	112.6	mm
Mean diameter	b_4	110.05	mm
Free height	h_0	7	mm
Band width	b_3	2.5	mm
Thickness	t_0	1.5	mm
Material	–	Polytetrafluoroethylene	
Young's modulus	E_1	0.28	GPa
Poisson's ratio	ν	0.4	–
Target axial load	–	3	N
Equivalent axial stiffness (FEA)	k_p	2.96	N/mm

Table S3: Data corresponding to Manuscript Table 3: finite-element stiffness comparison.

Object	Axial load (N)	Maximum deformation (mm)	Equivalent stiffness (N/mm)
Preload mechanism	3	1.0081	2.96
Three overlapping blades	3	0.13387	22.4

Table S4: Data corresponding to Manuscript Table 4: control factors and levels for the Taguchi design (unit: mm).

Factor	Level 1	Level 2	Level 3	Level 4	Level 5
L_1	1.9	2.0	2.1	2.2	2.3
L_2	15.1	15.2	15.3	15.4	15.5
R_1	9.3	9.4	9.5	9.6	9.7
R_2	1.2	1.3	1.4	1.5	1.6
T	3.2	3.3	3.4	3.5	3.6

Table S5: Data corresponding to Manuscript Table 5: $L_{25}(5^5)$ orthogonal array and confirmation results.

Run	L_1 (mm)	L_2 (mm)	R_1 (mm)	R_2 (mm)	T (mm)	Δf (Hz)
1	1.9	15.1	9.3	1.2	3.2	2160
2	1.9	15.2	9.4	1.3	3.3	1660
3	1.9	15.3	9.5	1.4	3.4	350
4	1.9	15.4	9.6	1.5	3.5	370
5	1.9	15.5	9.7	1.6	3.6	840
6	2.0	15.1	9.4	1.4	3.5	970
7	2.0	15.2	9.5	1.5	3.6	440
8	2.0	15.3	9.6	1.6	3.2	720
9	2.0	15.4	9.7	1.2	3.3	1110
10	2.0	15.5	9.3	1.3	3.4	1180
11	2.1	15.1	9.5	1.6	3.3	360
12	2.1	15.2	9.6	1.2	3.4	960
13	2.1	15.3	9.7	1.3	3.5	470
14	2.1	15.4	9.3	1.4	3.6	470
15	2.1	15.5	9.4	1.5	3.2	380
16	2.2	15.1	9.6	1.3	3.6	390
17	2.2	15.2	9.7	1.4	3.2	350
18	2.2	15.3	9.3	1.5	3.3	1010
19	2.2	15.4	9.4	1.6	3.4	640
20	2.2	15.5	9.5	1.2	3.5	1340
21	2.3	15.1	9.7	1.5	3.4	750
22	2.3	15.2	9.3	1.6	3.5	820
23	2.3	15.3	9.4	1.2	3.6	1300
24	2.3	15.4	9.5	1.3	3.2	730
25	2.3	15.5	9.6	1.4	3.3	380
Confirmation	2.1	15.3	9.5	1.5	3.3	310

Table S6: Data corresponding to Manuscript Table 6: design variables and predicted driving-tip amplitudes of the optimization candidates.

Candidate	d_1 (mm)	d_2 (mm)	d_3 (mm)	d_4 (mm)	d_5 (°)	Tangential (μm)	Normal (μm)
1	2.8	3.2	1.3	1.4	135.7	2881.4	1064.5
2	2.9	3.7	1.4	1.5	135.9	1620.8	982.6
3	3.2	3.7	1.4	1.4	135.8	705.8	576.7

Table S7: Data corresponding to Manuscript Table 7: modal verification of the flexible-clamping candidates.

Candidate	Adjacent 1 (kHz)	Working 1 (kHz)	Working 2 (kHz)	Adjacent 2 (kHz)	Minimum separation (kHz)
1	118.90	121.96	122.60	142.77	3.06
2	103.53	121.96	122.21	133.90	11.69
3	105.96	121.93	122.10	136.93	14.83

Table S8: Data corresponding to Manuscript Table 8: statistical ranges of the electrical characteristics of ten stators.

Metric	Phase-A range	Phase-B range	Principal observation
Static capacitance (nF)	2.113–2.209	2.120–2.286	Concentrated near 2.2 nF
Series-resonance frequency (Hz)	118,972–120,098	118,990–120,233	Similar between phases
Parallel-resonance frequency (Hz)	120,060–122,240	119,960–122,240	Larger deviations in individual samples

Table S9: Data corresponding to Manuscript Table 9: simulated and measured working-mode frequencies.

Source	Symmetric mode (kHz)	Antisymmetric mode (kHz)	Separation (Hz)
Finite-element analysis	122.10	121.93	170
Experiment	121.50	120.70	800
Relative frequency deviation	0.5%	1.0%	–

Table S10: Data corresponding to Manuscript Table 10: load performance of the V-shaped ultrasonic motor.

Mass (g)	Max. right (N)	Max. left (N)	Mean right (N)	Mean left (N)	Max. ratio (N/kg)
5.74	4.11	3.91	4.03	3.87	716

Table S11: Data corresponding to Manuscript Table 11: identified parameters of the direction-specific grey-box models.

Direction	g	p	b	q
Opening	70.38	111.52	0.0200	-43.14
Closing	-6.19	-15.85	0.0035	6.67

Table S12: Data corresponding to Manuscript Table 12: open-loop regulation performance over the tested local aperture range.

Direction	Model fit (%)	Angular-error range (°)	MAE (°)
Opening	95.46	-0.175–0.144	0.087
Closing	91.28	-0.286–0.246	0.13

2 Point-by-Point Data for Data-Bearing Figures

2.1 Manuscript Figure 14: Stepwise Displacement Response

Table S13: Point-by-point data corresponding to Manuscript Figure 14.

Time (ms)	Displacement (mm)
0	0.000531
0.1900238	0.000531
0.3800475	0.000531
0.5700713	0.0005752
0.760095	0.000531
0.9501188	0.0006195
1.1401425	0.0006637
1.3301663	0.0006637
1.52019	0.0006195
1.7102138	0.0006195
1.9002375	0.0005752
2.0902613	0.0004867
2.280285	0.0004425
2.4703088	0.0003982
2.6603325	0.0003982
2.8503563	0.0003982
3.04038	0.0003097
3.2304038	0.0002655
3.4204276	0.0003097
3.6104513	0.0003097
3.8004751	0.0002655
3.9904988	0.0002655
4.1805226	0.0004425
4.3705463	0.000531
4.5605701	0.0005752
4.7505938	0.0006195
4.9406176	0.0007522
5.1306413	0.0008407
5.3206651	0.000885

Continued on next page

Table S13 (continued)

Time (ms)	Displacement (mm)
5.5106888	0.0009292
5.7007126	0.000885
5.8907363	0.0008407
6.0807601	0.0007522
6.2707838	0.000708
6.4608076	0.0006195
6.6508314	0.0004867
6.8408551	0.0004425
7.0308789	0.000354
7.2209026	0.0003982
7.4109264	0.000531
7.6009501	0.0006195
7.7909739	0.000708
7.9809976	0.0007965
8.1710214	0.000885
8.3610451	0.0008407
8.5510689	0.0008407
8.7410926	0.0007522
8.9311164	0.000708
9.1211401	0.0006637
9.3111639	0.0006637
9.5011876	0.0007522
9.6912114	0.0006637
9.8812352	0.0006637
10.0712589	0.0011947
10.2612827	0.0030088
10.4513064	0.0037611
10.6413302	0.0053982
10.8313539	0.0052212
11.0213777	0.0053097
11.2114014	0.0053097
11.4014252	0.0053097
11.5914489	0.0052655
11.7814727	0.0052655
11.9714964	0.0053097
12.1615202	0.0053982
12.3515439	0.0053097
12.5415677	0.0052655
12.7315914	0.0053097
12.9216152	0.0052655
13.111639	0.0052212
13.3016627	0.0052212
13.4916865	0.0051327
13.6817102	0.0051327
13.871734	0.0052212
14.0617577	0.0051327
14.2517815	0.0050885
14.4418052	0.0052212
14.631829	0.0053097
14.8218527	0.0052212
15.0118765	0.0052212
15.2019002	0.0052212
15.391924	0.0051327
15.5819477	0.0050885
15.7719715	0.0050885
15.9619952	0.0051327
16.152019	0.0050885
16.3420428	0.0050442
16.5320665	0.0050442
16.7220903	0.0050442
16.912114	0.005
17.1021378	0.0050442
17.2921615	0.0050885
17.4821853	0.005177
17.672209	0.0050885
17.8622328	0.005
18.0522565	0.0050442
18.2422803	0.0050885
18.432304	0.0050442
18.6223278	0.0050442
18.8123515	0.0050442
19.0023753	0.0050442
19.192399	0.0050442
19.3824228	0.0050442
19.5724466	0.0050885
19.7624703	0.0050442
19.9524941	0.005
20.1425178	0.0050442

Continued on next page

Table S13 (continued)

Time (ms)	Displacement (mm)
20.3325416	0.0050442
20.5225653	0.0050442
20.7125891	0.005
20.9026128	0.0049558
21.0926366	0.005
21.2826603	0.005
21.4726841	0.0049558
21.6627078	0.0049558
21.8527316	0.005
22.0427553	0.0050442
22.2327791	0.0069469
22.4228029	0.0072566
22.6128266	0.0091593
22.8028504	0.0092035
22.9928741	0.0091593
23.1828979	0.009115
23.3729216	0.0090708
23.5629454	0.0090708
23.7529691	0.0090708
23.9429929	0.0090265
24.1330166	0.0090708
24.3230404	0.0090265
24.5130641	0.0090708
24.7030879	0.0090708
24.8931116	0.009115
25.0831354	0.0090708
25.2731591	0.0090265
25.4631829	0.0090708
25.6532067	0.0091593
25.8432304	0.0091593
26.0332542	0.0092035
26.2232779	0.0092035
26.4133017	0.0092478
26.6033254	0.0092035
26.7933492	0.0092035
26.9833729	0.0092478
27.1733967	0.0092035
27.3634204	0.0092035
27.5534442	0.0092035
27.7434679	0.0092478
27.9334917	0.0092035
28.1235154	0.0091593
28.3135392	0.0092035
28.5035629	0.0092035
28.6935867	0.009115
28.8836105	0.0091593
29.0736342	0.0091593
29.263658	0.0091593
29.4536817	0.0091593
29.6437055	0.0090708
29.8337292	0.009115
30.023753	0.0090708
30.2137767	0.0090708
30.4038005	0.009115
30.5938242	0.0092035
30.783848	0.0091593
30.9738717	0.009115
31.1638955	0.0091593
31.3539192	0.0091593
31.543943	0.009115
31.7339667	0.0092035
31.9239905	0.0092478
32.1140143	0.0092035
32.304038	0.0092035
32.4940618	0.0092035
32.6840855	0.0092035
32.8741093	0.009115
33.064133	0.0091593
33.2541568	0.009115
33.4441805	0.0091593
33.6342043	0.0092035
33.824228	0.0092035
34.0142518	0.009115
34.2042755	0.009646
34.3942993	0.0110177
34.584323	0.0115487
34.7743468	0.0128319
34.9643705	0.0128319

Continued on next page

Table S13 (continued)

Time (ms)	Displacement (mm)
35.1543943	0.0128319
35.3444181	0.0128319
35.5344418	0.0128761
35.7244656	0.0128761
35.9144893	0.0128761
36.1045131	0.0128319
36.2945368	0.0128319
36.4845606	0.0128761
36.6745843	0.0129204
36.8646081	0.0129646
37.0546318	0.0129204
37.2446556	0.0129204
37.4346793	0.0129646
37.6247031	0.0129646
37.8147268	0.0130088
38.0047506	0.0130088
38.1947743	0.0129646
38.3847981	0.0129204
38.5748219	0.0128319
38.7648456	0.0128319
38.9548694	0.0128319
39.1448931	0.0127876
39.3349169	0.0127876
39.5249406	0.0128319
39.7149644	0.0128319
39.9049881	0.0128319
40.0950119	0.0128319
40.2850356	0.0128319
40.4750594	0.0128761
40.6650831	0.0128319
40.8551069	0.0127876
41.0451306	0.0128319
41.2351544	0.0128319
41.4251781	0.0129646
41.6152019	0.0129204
41.8052257	0.0128761
41.9952494	0.0129204
42.1852732	0.0128319
42.3752969	0.0128761
42.5653207	0.0129204
42.7553444	0.0128319
42.9453682	0.0128761
43.1353919	0.0129204
43.3254157	0.0128761
43.5154394	0.0129204
43.7054632	0.0128761
43.8954869	0.0128319
44.0855107	0.0129204
44.2755344	0.0129204
44.4655582	0.0129646
44.6555819	0.0129204
44.8456057	0.0128761
45.0356295	0.0128761
45.2256532	0.0128319
45.415677	0.0127876
45.6057007	0.0127876
45.7957245	0.0128319
45.9857482	0.0127876
46.175772	0.0127876
46.3657957	0.014469
46.5558195	0.0147788
46.7458432	0.0164602
46.935867	0.0164159
47.1258907	0.0165044
47.3159145	0.0165044
47.5059382	0.0164602
47.695962	0.0164602
47.8859857	0.0164159
48.0760095	0.0163717
48.2660333	0.0163274
48.456057	0.0163717
48.6460808	0.0163274
48.8361045	0.0163717
49.0261283	0.0164602
49.216152	0.0164159
49.4061758	0.0163717
49.5961995	0.0164602
49.7862233	0.0164602

Continued on next page

Table S13 (continued)

Time (ms)	Displacement (mm)
49.976247	0.0164602
50.1662708	0.0164602
50.3562945	0.0163717
50.5463183	0.0162832
50.736342	0.0161947
50.9263658	0.0160619
51.1163895	0.0159735
51.3064133	0.0159735
51.4964371	0.0160619
51.6864608	0.0161062
51.8764846	0.0159735
52.0665083	0.0160177
52.2565321	0.0160619
52.4465558	0.0161062
52.6365796	0.0161504
52.8266033	0.0161504
53.0166271	0.0161504
53.2066508	0.0163274
53.3966746	0.0163717
53.5866983	0.0164602
53.7767221	0.0164159
53.9667458	0.0163274
54.1567696	0.0162832
54.3467933	0.0163717
54.5368171	0.0164159
54.7268409	0.0164159
54.9168646	0.0164602
55.1068884	0.0163717
55.2969121	0.0163717
55.4869359	0.0162389
55.6769596	0.0162389
55.8669834	0.0163717
56.0570071	0.0163274
56.2470309	0.0163717
56.4370546	0.0164159
56.6270784	0.0163717
56.8171021	0.0162832
57.0071259	0.0162832
57.1971496	0.0162832
57.3871734	0.0163717
57.5771971	0.0164159
57.7672209	0.0164602
57.9572447	0.0164602
58.1472684	0.0164159
58.3372922	0.0170796
58.5273159	0.0183628
58.7173397	0.019115
58.9073634	0.0203097
59.0973872	0.0203097
59.2874109	0.0202212
59.4774347	0.0202212
59.6674584	0.0202655
59.8574822	0.0202212
60.0475059	0.020177
60.2375297	0.020177
60.4275534	0.0201327
60.6175772	0.0200885
60.807601	0.0202212
60.9976247	0.0200885
61.1876485	0.0200442
61.3776722	0.0201327
61.567696	0.0200885
61.7577197	0.0200885
61.9477435	0.020177
62.1377672	0.020177
62.327791	0.0202655
62.5178147	0.0202655
62.7078385	0.0202655
62.8978622	0.0202212
63.087886	0.0203097
63.2779097	0.0203097
63.4679335	0.0202212
63.6579572	0.020177
63.847981	0.0202655
64.0380048	0.020354
64.2280285	0.020354
64.4180523	0.0202655
64.608076	0.0203097

Continued on next page

Table S13 (continued)

Time (ms)	Displacement (mm)
64.7980998	0.0202655
64.9881235	0.020354
65.1781473	0.0204425
65.368171	0.020354
65.5581948	0.0203097
65.7482185	0.0203097
65.9382423	0.020354
66.128266	0.0203982
66.3182898	0.020354
66.5083135	0.0203097
66.6983373	0.020354
66.888361	0.020354
67.0783848	0.0203097
67.2684086	0.0202655
67.4584323	0.0203097
67.6484561	0.020354
67.8384798	0.020354
68.0285036	0.020354
68.2185273	0.0203097
68.4085511	0.020354
68.5985748	0.0204425
68.7885986	0.0204425
68.9786223	0.0203982
69.1686461	0.0203982
69.3586698	0.0203982
69.5486936	0.0203982
69.7387173	0.020354
69.9287411	0.0203097
70.1187648	0.020354
70.3087886	0.0204867
70.4988124	0.0219912
70.6888361	0.0223009
70.8788599	0.0238053
71.0688836	0.0239381
71.2589074	0.0238496
71.4489311	0.0239381
71.6389549	0.0238938
71.8289786	0.0239823
72.0190024	0.0239823
72.2090261	0.0238938
72.3990499	0.0239381
72.5890736	0.0239381
72.7790974	0.0238938
72.9691211	0.0238938
73.1591449	0.0238938
73.3491686	0.0238938
73.5391924	0.0238938
73.7292162	0.0238053
73.9192399	0.0238053
74.1092637	0.0238496
74.2992874	0.0237611
74.4893112	0.0237168
74.6793349	0.0236726
74.8693587	0.0237168
75.0593824	0.0236726
75.2494062	0.0236726
75.4394299	0.0236726
75.6294537	0.0236726
75.8194774	0.0238053
76.0095012	0.0237168
76.1995249	0.0236283
76.3895487	0.0237611
76.5795724	0.0239381
76.7695962	0.0238496
76.95962	0.0239381
77.1496437	0.0239823
77.3396675	0.0239823
77.5296912	0.0239823
77.719715	0.0239823
77.9097387	0.0239823
78.0997625	0.0239823
78.2897862	0.0239823
78.47981	0.0239823
78.6698337	0.0239823
78.8598575	0.0239823
79.0498812	0.0239823
79.239905	0.0239823
79.4299287	0.0239823

Continued on next page

Table S13 (continued)

Time (ms)	Displacement (mm)
79.6199525	0.0239823
79.8099762	0.0239823
80	0.0239823

2.2 Manuscript Figure 15: Temperature-Dependent Characteristics

Table S14: Temperature, resonance frequency, and output-force data corresponding to Manuscript Figure 15.

Temperature (°C)	Resonance frequency (kHz)	Output force (N)
-10	120.457	0.22
0	120.134	0.76
10	119.864	1.09
20	119.413	2.08
25	119.185	3.23
30	118.965	1.74
40	118.513	0.56
50	118.054	0

2.3 Manuscript Figure 18: Drive-Response Characterization

Table S15: Rotation angle versus drive-cycle count corresponding to Manuscript Figure 18.

Drive cycles	Rotation angle (°)
100	0.06
150	0.089
200	0.179
250	0.268
300	0.357
350	0.476
400	0.595
450	0.685
500	0.804
550	0.893
600	1.012
650	1.042
700	1.31
750	1.458
800	1.607
850	1.786
900	1.875
950	1.994
1000	2.113
1050	2.202
1100	2.411
1150	2.53
1200	2.649
1250	2.708
1300	2.857
1350	2.976
1400	3.095
1450	3.333
1500	3.423

Table S16: Rotation angle versus drive voltage corresponding to Manuscript Figure 18.

Voltage (V_{pp})	Rotation angle (°)
50	0.32
75	0.58
100	0.9
125	1.18
150	1.4
175	1.7
200	1.82

Table S17: Rotation angle versus drive frequency corresponding to Manuscript Figure 18.

Frequency (kHz)	Rotation angle (°)
121.6	0.35
121.7	1.1
121.8	1.95
121.9	1.1
122	0.4

2.4 Manuscript Figure 19: Direction-Specific Identification

Table S18: Opening identification data corresponding to Manuscript Figure 19.

Drive cycles	Measured angle (°)	Model angle (°)	Residual (°)
100	0.058	0.008	-0.05
150	0.117	0.067	-0.05
200	0.214	0.194	-0.02
250	0.272	0.272	0
300	0.379	0.379	0
350	0.485	0.485	0
400	0.602	0.602	0
450	0.699	0.719	0.02
500	0.816	0.836	0.02
550	0.913	0.953	0.04
600	1.019	1.099	0.08
650	1.058	1.208	0.15
700	1.335	1.21729	-0.11771
750	1.476	1.426	-0.05
800	1.621	1.561	-0.06
850	1.816	1.736	-0.08
900	1.903	1.783	-0.12
950	2.029	1.929	-0.1
1000	2.146	2.066	-0.08
1050	2.233	2.183	-0.05
1100	2.437	2.417	-0.02
1150	2.524	2.474	-0.05
1200	2.66	2.64	-0.02
1250	2.728	2.778	0.05
1300	2.903	2.903	0
1350	3.01	3.06	0.05
1400	3.141	3.221	0.08
1450	3.369	3.349	-0.02
1500	3.466	3.496	0.03

Table S19: Closing identification data corresponding to Manuscript Figure 19.

Drive cycles	Measured angle (°)	Model angle (°)	Residual (°)
100	0.019	0.039	0.02
150	0.039	0.019	-0.02
200	0.085	0.055	-0.03
250	0.108	0.108	0
300	0.169	0.169	0
350	0.238	0.218	-0.02
400	0.369	0.299	-0.07
450	0.438	0.358	-0.08
500	0.508	0.428	-0.08
550	0.523	0.503	-0.02
600	0.546	0.576	0.03
650	0.715	0.665	-0.05
700	0.831	0.72857	-0.10243
750	0.896	0.796	-0.1
800	0.931	0.881	-0.05
850	0.969	0.889	-0.08
900	1.1	1.18	0.08
950	1.2	1.32	0.12
1000	1.277	1.317	0.04
1050	1.369	1.489	0.12
1100	1.462	1.622	0.16
1150	1.55	1.65	0.1
1200	1.8	1.98563	0.18563
1250	2	2.08	0.08
1300	2.192	2.172	-0.02
1350	2.346	2.306	-0.04
1400	2.562	2.542	-0.02
1450	2.785	2.785	0
1500	2.78	2.88	0.1

2.5 Manuscript Figure 20: Residual Correlation Analysis

Table S20: Opening residual-correlation data corresponding to Manuscript Figure 20.

Lag	Autocorrelation	Cross-correlation
-25	-0.04	0.01
-24	-0.03	0.03
-23	-0.02	0.02
-22	-0.01	-0.02
-21	0.03	-0.06
-20	0.04	-0.18
-19	0.01	-0.27
-18	-0.15	-0.22
-17	0.03	-0.15
-16	-0.1	-0.08
-15	0.02	-0.02
-14	0.18	0.04
-13	0.12	0.07
-12	-0.08	0.1
-11	-0.15	0.09
-10	-0.1	0.06
-9	-0.18	0.02
-8	-0.25	-0.03
-7	-0.13	-0.06
-6	-0.2	-0.07
-5	-0.05	-0.05
-4	0.1	-0.02
-3	0.12	0
-2	0.1	0.01
-1	0.1	0.02
0	1	0.02
1	0.1	0.02
2	0.1	0.01
3	0.12	0
4	0.1	-0.01
5	-0.05	-0.01
6	-0.2	-0.01
7	-0.13	0
8	-0.25	0.01
9	-0.18	0.01
10	-0.1	0.01
11	-0.15	0
12	-0.08	-0.01
13	0.12	-0.01
14	0.18	0
15	0.02	0.01
16	-0.1	0.01
17	0.03	0
18	-0.15	0
19	0.01	-0.01
20	0.04	0
21	0.03	0.01
22	-0.01	0
23	-0.02	0
24	-0.03	0
25	-0.04	0

Table S21: Closing residual-correlation data corresponding to Manuscript Figure 20.

Lag	Autocorrelation	Cross-correlation
-25	0.03	0.08
-24	0.06	0.1
-23	-0.08	0.04
-22	-0.1	-0.1
-21	-0.04	-0.12
-20	0.16	-0.06
-19	-0.12	0.02
-18	-0.1	0.08
-17	-0.05	0.04
-16	-0.03	-0.05
-15	0.29	-0.1
-14	0.18	0.06
-13	-0.28	0.1
-12	-0.12	0.05
-11	0.2	-0.04
-10	-0.3	-0.09
-9	0.12	-0.07

Continued on next page

Table S21 (continued)

Lag	Autocorrelation	Cross-correlation
-8	0.26	-0.04
-7	-0.18	-0.02
-6	-0.42	0.02
-5	-0.3	0.03
-4	-0.45	0.02
-3	-0.1	0.01
-2	0.1	0
-1	0.12	0
0	1	0
1	0.12	0
2	0.1	0
3	-0.1	0
4	-0.45	0
5	-0.3	0
6	-0.42	0
7	-0.18	0
8	0.26	0
9	0.12	0
10	-0.3	0
11	0.2	0
12	-0.12	0
13	-0.28	0
14	0.18	0
15	0.29	0
16	-0.03	0
17	-0.05	0
18	-0.1	0
19	-0.12	0
20	0.16	0
21	-0.04	0
22	-0.1	0
23	-0.08	0
24	0.06	0
25	0.03	0

2.6 Manuscript Figure 21: Open-Loop Regulation Results

Table S22: Opening regulation data corresponding to Manuscript Figure 21.

Trial	Measured angle (°)	Error (°)	Absolute error (°)
1	9.872	0.0394	0.0394
2	9.606	-0.175	0.175
3	9.901	0.123	0.123
4	9.931	0.144	0.144
5	9.783	-0.0472	0.0472
6	9.901	0.0745	0.0745
7	9.842	0.0616	0.0616
8	9.872	0.046	0.046
9	9.754	-0.0774	0.0774
10	9.724	-0.0654	0.0654
11	9.665	-0.167	0.167
12	9.754	-0.0716	0.0716
13	9.783	-0.0502	0.0502

Table S23: Closing regulation data corresponding to Manuscript Figure 21.

Trial	Measured angle (°)	Error (°)	Absolute error (°)
1	10.217	0.155	0.155
2	10.217	0.129	0.129
3	10.065	0.0256	0.0256
4	9.945	-0.142	0.142
5	9.975	-0.0655	0.0655
6	9.975	-0.117	0.117
7	9.824	-0.256	0.256
8	9.884	-0.153	0.153
9	10.126	0.0337	0.0337
10	10.2	0.246	0.246
11	9.793	-0.286	0.286
12	10.035	-0.0531	0.0531
13	10.156	0.0644	0.0644

2.7 Manuscript Figure 22: Full-Range Resistance and Model Extrapolation

Table S24: Raw repeated measurements of equivalent tangential resistance corresponding to Manuscript Figure 22(a).

Aperture (mm)	Direction	Replicate	Equivalent resistance (N)
6	Opening	1	0.78
6	Opening	2	0.75
6	Opening	3	0.72
6	Closing	1	0.85
6	Closing	2	0.87
6	Closing	3	0.81
8	Opening	1	0.61
8	Opening	2	0.65
8	Opening	3	0.6
8	Closing	1	0.64
8	Closing	2	0.69
8	Closing	3	0.65
10	Opening	1	0.54
10	Opening	2	0.53
10	Opening	3	0.51
10	Closing	1	0.51
10	Closing	2	0.55
10	Closing	3	0.49
20	Opening	1	0.51
20	Opening	2	0.53
20	Opening	3	0.56
20	Closing	1	0.58
20	Closing	2	0.54
20	Closing	3	0.51
40	Opening	1	0.49
40	Opening	2	0.46
40	Opening	3	0.52
40	Closing	1	0.51
40	Closing	2	0.57
40	Closing	3	0.51
60	Opening	1	0.53
60	Opening	2	0.49
60	Opening	3	0.5
60	Closing	1	0.52
60	Closing	2	0.55
60	Closing	3	0.5
80	Opening	1	0.53
80	Opening	2	0.52
80	Opening	3	0.51
80	Closing	1	0.56
80	Closing	2	0.52
80	Closing	3	0.61
90	Opening	1	0.62
90	Opening	2	0.61
90	Opening	3	0.59
90	Closing	1	0.58
90	Closing	2	0.56
90	Closing	3	0.59
95	Opening	1	0.78
95	Opening	2	0.71
95	Opening	3	0.73
95	Closing	1	0.65
95	Closing	2	0.67
95	Closing	3	0.63

Table S25: Target and measured apertures corresponding to Manuscript Figure 22(b).

Target (mm)	Measured (mm)	Direction	Error (mm)	Note
5.5	7.2	Closing	1.7	–
6	7.5	Closing	1.5	–
8	8.5	Closing	0.5	–
10	10.1	Closing	0.1	–
15	14.8	Closing	-0.2	–
20	20	Reference	0	20 mm baseline
30	30.2	Opening	0.2	–
50	50.1	Opening	0.1	–
70	69.8	Opening	-0.2	–
80	80.3	Opening	0.3	–
90	90.4	Opening	0.4	–
98	96.8	Opening	-1.2	–

3 Key Numerical Results

Table S26: Summary of the principal numerical results in the accepted manuscript.

Result	Value	Unit
Preload/blade-stack stiffness ratio	7.6	–
Simulated working-mode separation	170	Hz
Measured working-mode separation	800	Hz
Maximum bidirectional thrusts	3.91 / 4.11	N
Maximum thrust-to-mass ratio	716	N/kg
Minimum observed stable tangential displacement	3.23	μm
Resonance-frequency decrease from -10 to 50 °C	2.403	kHz
Maximum output force in the temperature test	3.23 at 25	N, °C
Opening mean absolute ring-angle error	0.087	°
Closing mean absolute ring-angle error	0.13	°
Local model-identification and regulation interval	10–20	mm
Full-range extrapolation MAE over 10–80 mm	0.183	mm
Endpoint aperture errors at 5.5 and 98 mm	+1.7 / -1.2	mm
Maximum mean quasi-static tangential resistance	0.843	N

4 File and Variable Guide

The source tables used to generate this document are retained with the LaTeX source. Variable names encode the reported quantity and unit. The direction labels “Opening”, “Closing”, and “Reference” follow the terminology of the accepted manuscript. No interpolation was applied to the tabulated point-by-point data.
